# Growth Factor-Induced Mobilization of Cardiac Progenitor Cells Reduces the Risk of Arrhythmias, in a Rat Model of Chronic Myocardial Infarction

**DOI:** 10.1371/journal.pone.0017750

**Published:** 2011-03-18

**Authors:** Leonardo Bocchi, Monia Savi, Gallia Graiani, Stefano Rossi, Aldo Agnetti, Francesca Stillitano, Costanza Lagrasta, Silvana Baruffi, Roberta Berni, Caterina Frati, Mario Vassalle, Umberto Squarcia, Elisabetta Cerbai, Emilio Macchi, Donatella Stilli, Federico Quaini, Ezio Musso

**Affiliations:** 1 Dipartimento di Biologia Evolutiva e Funzionale, Università di Parma, Parma, Italy; 2 Dipartimento di Patologia e Medicina di Laboratorio, Università di Parma, Parma, Italy; 3 Dipartimento dell'Età Evolutiva, Università di Parma, Parma, Italy; 4 Dipartimento di Farmacologia Preclinica e Clinica, Università di Firenze, Firenze, Italy; 5 Department of Physiology and Pharmacology, SUNY, Downstate Medical Center, Brooklyn, New York, United States of America; 6 Dipartimento di Medicina Interna e Scienze Biomediche, Università di Parma, Parma, Italy; 7 Centro Interdipartimentale Cellule Staminali Cardiache (CISTAC), Università di Parma, Parma, Italy; Brigham and Women's Hospital, United States of America

## Abstract

Heart repair by stem cell treatment may involve life-threatening arrhythmias. Cardiac progenitor cells (CPCs) appear best suited for reconstituting lost myocardium without posing arrhythmic risks, being commissioned towards cardiac phenotype. In this study we tested the hypothesis that mobilization of CPCs through locally delivered Hepatocyte Growth Factor and Insulin-Like Growth Factor-1 to heal chronic myocardial infarction (MI), lowers the proneness to arrhythmias. We used 133 adult male Wistar rats either with one-month old MI and treated with growth factors (GFs, n = 60) or vehicle (V, n = 55), or sham operated (n = 18). In selected groups of animals, prior to and two weeks after GF/V delivery, we evaluated stress-induced ventricular arrhythmias by telemetry-ECG, cardiac mechanics by echocardiography, and ventricular excitability, conduction velocity and refractoriness by epicardial multiple-lead recording. Invasive hemodynamic measurements were performed before sacrifice and eventually the hearts were subjected to anatomical, morphometric, immunohistochemical, and molecular biology analyses. When compared with untreated MI, GFs decreased stress-induced arrhythmias and concurrently prolonged the effective refractory period (ERP) without affecting neither the duration of ventricular repolarization, as suggested by measurements of QTc interval and mRNA levels for K-channel α-subunits Kv4.2 and Kv4.3, nor the dispersion of refractoriness. Further, markers of cardiomyocyte reactive hypertrophy, including mRNA levels for K-channel α-subunit Kv1.4 and β-subunit KChIP2, interstitial fibrosis and negative structural remodeling were significantly reduced in peri-infarcted/remote ventricular myocardium. Finally, analyses of BrdU incorporation and distribution of connexin43 and N-cadherin indicated that cytokines generated new vessels and electromechanically-connected myocytes and abolished the correlation of infarct size with deterioration of mechanical function. In conclusion, local injection of GFs ameliorates electromechanical competence in chronic MI. Reduced arrhythmogenesis is attributable to prolongation of ERP resulting from improved intercellular coupling via increased expression of connexin43, and attenuation of unfavorable remodeling.

## Introduction

During the last decade, a growing number of experimental and clinical observations have raised the possibility of treating cardiac damage through the direct repairing ability or the developmental plasticity of stem cells [1]. Although not generally agreed, therapies based on the mobilization/injection of several types of stem cells have been shown to ameliorate the mechanical function and perfusion in ischemic cardiomyopathy [2]–[4].

A few clinical and experimental studies have addressed the issue of the electrophysiological effects of implantation, homing, engraftment and differentiation of regenerating cells within the damaged myocardium [5]–[9]. The improvement of cardiac mechanical function by stem cell based treatments can be associated with increase, reduction or no changes in the risk of arrhythmias [5]–[9]. Often, the occurrence of arrhythmias is negatively correlated with the ability of the newly formed tissue to adopt a cardiac fate or to couple electrically with the spared myocardium [10]–[15].

The heart contains several cohorts of resident cardiac progenitor cells (CPCs) which have been characterized by different groups [16]–[22] and are considered to be responsible for tissue homeostasis [23]. Because CPCs are intrinsically programmed to generate myocardium, they appear best suited for the complex task of reconstituting tissue that is lost with a myocardial infarction [24] and restoring the blood supply in the damaged area [25]. These properties of CPCs would be expected to improve the organization and functional integration of newly formed tissues with the spared tissue, minimizing heterogeneous electrical remodeling and poor intercellular coupling occurring with numerous cardiac regenerative treatments [10]–[15]. Thus, mending of the heart promoted by CPCs can be an appropriate substrate to explore the proarrhythmic or antiarrhythmic consequences of stem cell based myocardial regeneration.

CPCs have been reported to express c-Met and insulin-like growth factor-1 (IGF-1) receptors and synthesize and secrete the corresponding ligands, hepatocyte growth factor (HGF) (which mobilizes CPCs) and IGF-1 (which promotes their survival and proliferation) [26]. In infarcted hearts of dogs [27], mice [26], and rats [28], the intramyocardial injection of HGF and IGF-1 enhanced the translocation of CPCs from the surrounding myocardium to the dead tissue and their viability and growth within the damaged area, fostering cardiac regeneration and improving mechanical function. In the present study, we tested the hypothesis that activating the lineage commitment and progeny formation of resident CPCs, via the HGF/IGF-1-receptor systems, can also ameliorate the electrical competence of the infarcted heart, in a rat model of chronic myocardial infarction (MI). Importantly, post-MI ventricular remodeling contributes to end stage heart failure [29], and lethal arrhythmias are responsible for up to half the deaths in heart failure [30].

By following an approach from the intact animal to tissue, cellular, and molecular levels, we found that repair of chronic MI by cytokine treatment significantly reduced the occurrence of arrhythmias. GFs also promoted the development of new myocardial tissue, attenuated negative structural remodeling, and partially restored mechanical function.

The amelioration in cardiac electrical stability appeared to include a prolongation of the effective refractory period without changes in the duration of the recovery process attributed to a better intercellular coupling, and positive ventricular remodeling.

## Methods

A detailed description of the methods is provided in the supporting information (File S1). This study was carried out in strict accordance with the recommendations in the Guide for the Care and Use of Laboratory Animals of the National Institute of Health. The protocol was approved by the Veterinary Animal Care and Use Committee of the University of Parma and conforms to the National Ethical Guidelines of the Italian Ministry of Health (Permit number: 41/2009-B).

All surgery was performed under anesthesia (ketamine+medetomidine or droperidol+fentanyl citrate), and all efforts were made to minimize suffering.

### Animal population

Two hundred and seven male Wistar rats, aged 12–14 weeks and weighing 300–350 g, were subjected either to myocardial infarction (MI group) or to sham operation (SO group). Four weeks later, all animals were treated with HGF+IGF-1 (GF) or vehicle (V) and assigned to the following subgroups: MI+GF, MI+V and SO+V. Fifty four rats died in the peri-operative periods and 20 additional rats, assigned to the MI+GF or MI+V groups, were excluded from the study because infarction could not be clearly detected at autopsy, leaving a total of 133 rats.

### Outline of the experimental protocols

The experimental design and measurements performed in the study are summarized in Table S1.

#### In vivo studies

The occurrence of spontaneous and stress-induced arrhythmias in conscious, freely moving rats was evaluated by Telemetry-ECG recording (TE rats, including 22 MI+GF, 23 MI+V, and 15 SO+V animals), prior to and 15 days after GF or V (GF/V) administration. At those times, cardiac mechanical function was also evaluated by echocardiography. Invasive hemodynamic measurements were performed before sacrifice. Simultaneously with GF/V injection, selected subgroups of TE rats (MI+GF: n = 14 and MI+V: n = 11), were treated with 5-Bromo-2′-deoxycytidine (BrdC) which in vivo is metabolically converted to BrdU (5-Bromo-2′-deoxyuridine), to evaluate the cumulative amount of cell proliferation. Conduction velocity, excitability, refractoriness, dispersion of refractoriness and QT interval duration were measured by Epicardial Multiple-lead recording (EM rats) in an additional 24 MI+GF and 19 MI+V animals.

#### Cardiac anatomy and morphometric analyses

Hearts obtained from TE and EM rats were perfusion fixed: (i) to ascertain myocardial infarction (all animals), and (ii) to evaluate Left Ventricular (LV) anatomical changes and infarct size, and to carry out morphometric and immunohistochemical analyses (selected subgroups of TE rats).

#### Molecular studies

In 30 additional animals, two weeks after GF/V injection the heart was excised and immediately frozen for Molecular Biology evaluation (MB rats) which included electrophoretic and immunoblot assays (5 MI+GF and 5 MI+V animals) and quantitative RT-PCR measurements (3 SO+V, 9 MI+GF and 8 MI+V animals).

### Telemetry-ECG data acquisition and processing and stress-induced sympathetic stimulation

A miniaturized telemetry-ECG transmitter was chronically implanted in TE rats under anesthesia, as previously described [31]. A 15-minute telemetry-ECG recording was performed in experimental animals when alone and undisturbed in their home cage (baseline period) and again when subjected to stress-induced sympathetic stimulation (stress period) [32]. Telemetry-ECGs were processed off-line for evaluating the incidence of ventricular arrhythmias, heart rate (1/R-R interval), and time domain indexes of heart rate variability (SD_RR_: standard deviation of average R-R interval; r-MSSD: root mean square of successive R-R interval square differences), as indirect measurements of the autonomic input to the heart [33].

### Myocardial infarction

In MI animals under anesthesia and artificial ventilation, a thoracotomy via the third left-intercostal space was performed and the left coronary artery was ligated. The chest was then closed, the pneumothorax was reduced by suction of air and fluid and the rats were allowed to recover. In SO rats the ligature around the coronary artery was not tied.

### Growth factor and BrdC administration

Four weeks after coronary artery ligature, a second left lateral thoracotomy was performed in all animals. In MI+GF rats, GFs were injected in six intramural sites: near the left atrium, between the atrium and the infarct and at four opposite sites of the border zone [26]. The concentration of IGF-1 was constant (200 ng/mL) whereas HGF was administered at increasing concentrations from the atrium to the border zone (50, 100, 200 ng/mL). In MI+V and SO+V rats, corresponding regions of the heart were injected with vehicle. In MI+GF animals, Rhodamine spheres were added to the solutions (v/v 5%) to mark the injection sites.

In BrdC treated rats, a continuous infusion of BrdC (0.6 mol/L) was performed until sacrifice (two weeks) by an osmotic pump implanted subcutaneously in the inter-scapular region. The long infusion time prompted us to use BrdC rather than the most commonly employed BrdU because of the nearly six-fold higher solubility of BrdC.

### Echocardiography

Serial echocardiograms were obtained from TE rats under anesthesia. Two-dimensional (2-D) and M-mode images were recorded from modified parasternal long axis and parasternal short axis views. Systolic and diastolic morpho-functional parameters were evaluated using standard methods [34].

### Invasive hemodynamics

In anesthetized TE rats, systolic and diastolic arterial blood pressures were recorded by means of a microtip pressure transducer inserted into the right carotid artery. The catheter was then advanced into the left ventricle to measure left ventricular systolic and end-diastolic pressures, and +/− dP/dt, in the closed-chest preparation.

### Epicardial multiple-lead recording

EM rats under anesthesia and artificial respiration were subjected to left thoracotomy. A 5×5 or 8×8 row and column electrode array [35] (see Fig. S1A–B), was positioned over the infarcted region and the surrounding areas. Epicardial unipolar electrograms (EGs) were recorded during sinus rhythm or specific pacing protocols in order to determine: (i) conduction velocity at the ventricular surface along and across fibers, (ii) cardiac excitability and refractoriness by strength duration curve (S-D curve) and effective refractory period (ERP) measurements respectively, (iii) dispersion of refractoriness [36], and (iv) duration of QT and corrected QT interval (QTc) [37]–[38].

### Cardiac anatomy

In each heart, after perfusion with 10% buffered formalin, the left and right ventricular weights and LV chamber volume were determined. The LV diameter and wall thickness were computed on the equatorial transverse section of the ventricle, cut perpendicularly to the major axis. The LV chamber volume was calculated according to the Dodge equation [39]. Subsequently, from the equatorial slice embedded in paraffin, five-micrometer thick sections were cut and used for morphometric and immunohistochemical analyses.

### Morphometric analysis

Infarct size was measured by calculating the fraction of myocytes lost as a result of coronary occlusion [40]–[41]. Myocardial sections stained with Masson's trichrome were analyzed by optical microscopy to assess the volume fraction of myocytes and perivascular and interstitial fibrosis, according to a procedure previously described [42].

### Immunohistochemical analysis

Sections from BrdC treated rat hearts with comparable infarct size were analyzed to determine: (i) the expression and spatial distribution of connexin43 (Cx43) and N-cadherin, (ii) the incidence of c-kit+ CPCs, and (iii) the fraction of nuclei labeled by BrdU, in the infarcted, peri-infarcted and remote LV myocardium. The amount of newly formed myocardium was computed by calculating: (i) the number and size of small α-sarcomeric actin positive (α-SARC+) myocytes and (ii) the number of resistance arterioles and capillaries labeled by α-smooth muscle actin (α-SMA) and von Willebrand Factor (vW) respectively, according to a methodology previously employed [41].

### Biochemical and Molecular Biology analyses

Immediately after death, left and right ventricles were excised from the heart of 30 MB rats (see Table S1), weighed and immediately frozen at −80°C.

#### Electrophoretic and immunoblot assay

The infarcted and non-infarcted portions of the left ventricles obtained from 5 MI+GF and 5 MI+V hearts were used for immunoblot assay of Cx43.

#### Quantification of Kv1.4, Kv4.2, Kv4.3 and KChIP2 transcripts

In 3 SO+V, 9 MI+GF and 8 MI+V animals, total RNA was harvested from frozen tissue samples using a column-based extraction method. Gene expression was evaluated by reverse transcription quantitative polymerase chain reaction (RT-qPCR). Real-Time PCR for Kv1.4, Kv4.2, Kv4.3 and KChIP2 was performed using an ABI Prism 7500 Sequence Detection System with TaqMan gene expression assays.

### Statistical analysis

The SPSS statistical package was used (SPSS, Chicago, IL, USA, 17th version). Normal distribution of variables was checked by means of the Kolmogorov-Smirnov test. Statistics of variables normally distributed (all variables except baseline arrhythmias) included mean ± standard error (SE), paired and unpaired Student's t test, and one-way analysis of variance (post-hoc analysis: Games-Howell test and Tukey test when appropriate). Non-parametric statistical tests were used to evaluate differences in the incidence of baseline ventricular arrhythmias among groups (Kruskall-Wallis test and Mann-Whitney U-test), and differences between baseline and stress-induced arrhythmias within each group (Wilcoxon test). Statistical significance was set at p<0.05.

## Results

### 1) Electrical function

#### Telemetry-ECG recording

R-R interval, SD_RR_ and r-MSSD during baseline ECG recordings had similar values in all groups (R-R: approximately 175 ms, on average; SD_RR:_ 11 ms; r-MSSD: 4 ms), before and after GF/V injection. Stress procedure generally increased heart rate (1/R-R interval) by about 30% and reduced SD_RR_ and r-MSSD by about 40% and 30% respectively (p<0.01), as a result of the enhanced sympathetic activity brought about by the social challenge [32]. The effects of stress were similar before and after GF/V injection in each animal group, suggesting that GFs did not affect the autonomic input to the regenerated heart.

**Figure 1:**
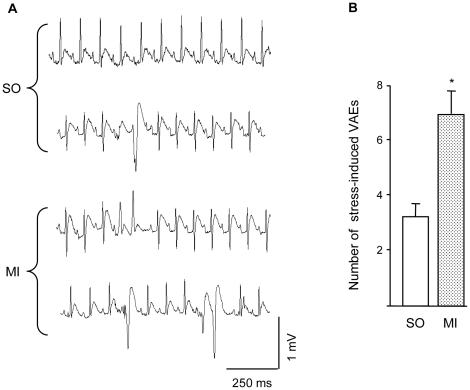
Ventricular arrhythmic events. A) Telemetry-ECG recordings during social stress from representative SO (upper tracings) and MI animals (lower tracings) showing the different types and severity of ventricular arrhythmic events (VAEs). B) Average values±SE of the number of VAEs occurring in SO and MI groups, during stress exposure. * p<0.01 vs. SO.

**Figure 2:**
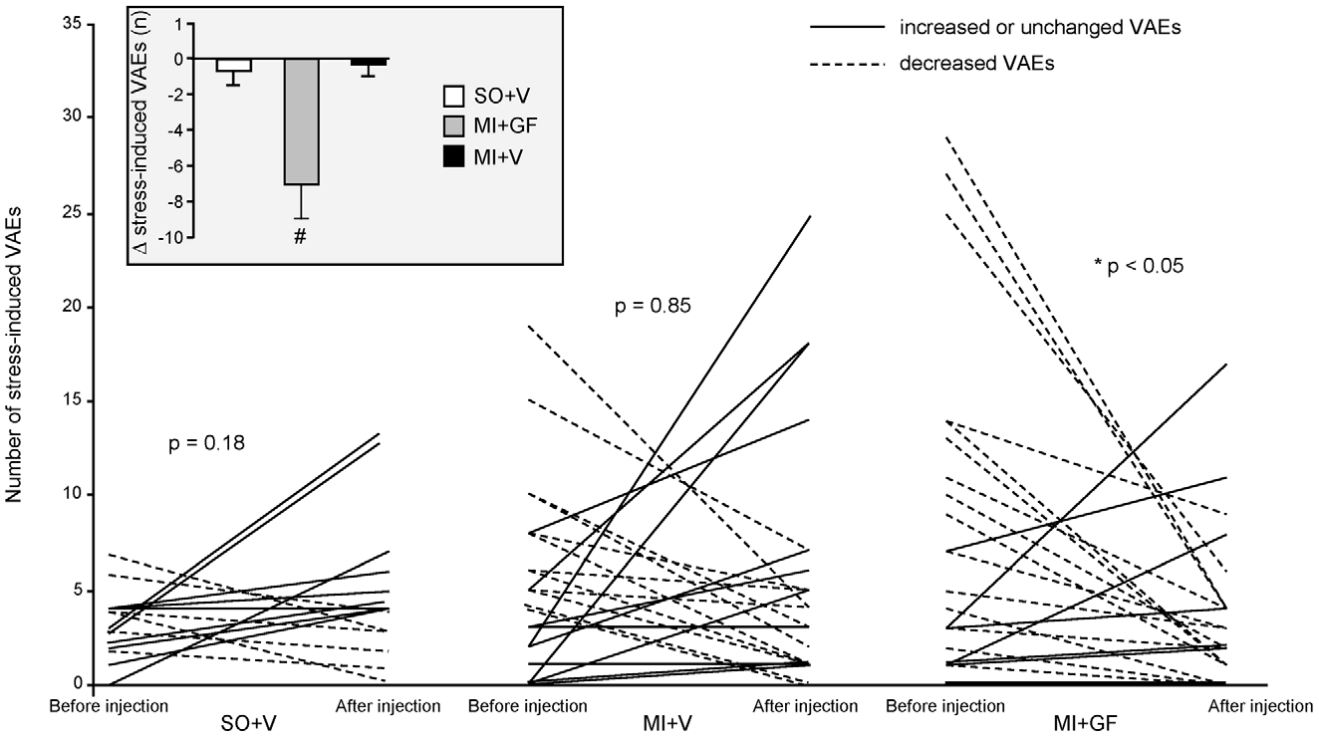
Effects of GF/V injection on the proneness to stress-induced arrhythmias. Changes in the number of stress-induced VAEs after GF/V injection, in each animal of SO+V, MI+V and MI+GF groups. * p<0.02 vs. pre-injection values, within MI+GF group. The insert shows the average values±SE of the difference (Δ) between the number of VAEs measured before and after GF/V injection, in each experimental group. # p<0.01 vs. SO+V and MI+V.

In all animals, ventricular arrhythmias mostly consisted of isolated premature beats and a few salvos. Arrhythmia vulnerability was evaluated as the number of ventricular arrhythmic events (VAEs) during the 15-minute baseline and stress periods. Before GF/V injection, VAEs were negligible during baseline in all groups (range 0–6 events). Stress increased VAEs in both SO and MI rats (p<0.01) although the increment was about two-fold higher in MI animals (p<0.05 vs. SO, Fig. 1A–B). Two weeks after injection, baseline-VAEs remained unchanged in all groups while stress-VAEs were markedly reduced in MI+GF rats (p<0.05) but not in SO+V and MI+V animals (Fig. 2). Thus, cytokine treatment lowered the proneness to arrhythmias triggered by stress-induced sympathetic stimulation in conscious animals with chronic myocardial infarction. Importantly, this procedure mimics stressful conditions encountered by social animals in everyday life.

#### Epicardial multiple lead recording

**Figure 3:**
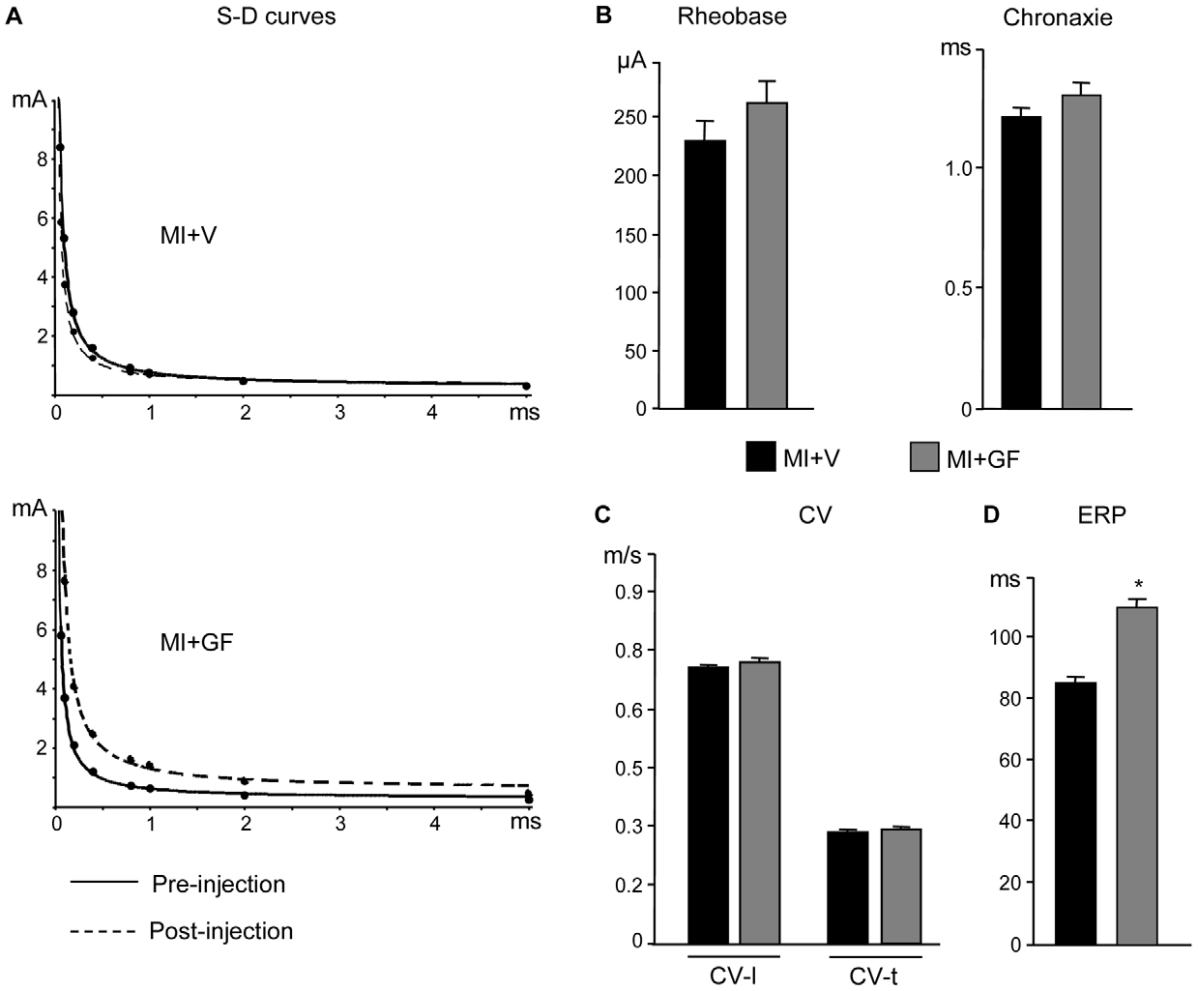
Epicardial multiple-lead recording. Strength-duration (S-D) curves (A) computed in representative rat hearts belonging to MI+V and MI+GF groups, before (solid line) and after (dotted line) GF/V injection. The bar graphs illustrate the mean values±SE of: (B) rheobase and chronaxie, (C) conduction velocity along (CV-l) and across (CV-t) epicardial fiber direction, and (D) effective refractory period (ERP), in MI+V and MI+GF groups. * p<0.001 vs. MI+V.

To measure excitability, 121, 120 and 114 Strength-Duration (S-D) curves and as many Rheobase (Rh) and Chronaxie (Chr) values (see Fig. S1F) were determined in the MI, MI+V and MI+GF groups respectively. Before injection, Rh and Chr in MI animals were 114±10 µA and 1.2±0.04 ms respectively. Cytokines shifted upward and to the right S-D curves and concurrently Rh and Chr values in MI+GF rats were slightly higher than those measured in MI+V group, suggesting a tendential reduction in excitability (Fig. 3A–B).

Given the limited number of electrodes of the 5×5 array, no reliable conduction velocity (CV) measurements could be performed in the MI rats and only post-injection data were collected in the MI+V and MI+GF groups. CV was computed longitudinally (CV-l) and transversally (CV-t) to fiber orientation (see Fig. S1E) at 114 and 112 epicardial sites in MI+V animals and 92 and 100 sites in MI+GF rats. The values of both CV-l and CV-t were similar in the two groups (Fig. 3C), indicating that growth factor administration did not have any sizable effect on the spread of excitation at the epicardial surface.

The effective refractory period (ERP) was measured at 87, 113 and 92 epicardial sites in the MI, MI+V and MI+GF animals respectively. In the MI rats the average ERP value was 88.8±2.4 ms. After GF/V injection, ERP was about 30% longer in the MI+GF animals when compared with the MI+V group (Fig. 3D, p<0.001). Interestingly, a longer ERP would reduce the probability of reentry circuits provided that CV increases or remains constant [43]. Thus, by the combined effect on conduction velocity and refractoriness, cytokine treatment is expected to reduce cardiac electrical instability. Indeed, stress induced VAEs were significantly reduced in GF treated animals (Fig. 2).

The degree of dispersion of ERP values and the duration of QTc interval were comparable in all animals (ERP dispersion: 11±1.5 ms in MI+V and 16±2.9 ms in MI+GF; QTc: 38.3±1 ms in MI+V and 39.4±12 ms in MI+GF) suggesting that (i) the influence of GFs on propensity to reentrant arrhythmias was not mediated by changes in the spatially non-uniform distribution of refractoriness and (ii) the longer ERP in MI+GF animals does not seem attributable to the longer duration of the recovery process.

#### Quantitative RT-PCR measurements of I_to_ current subunits

**Figure 4:**
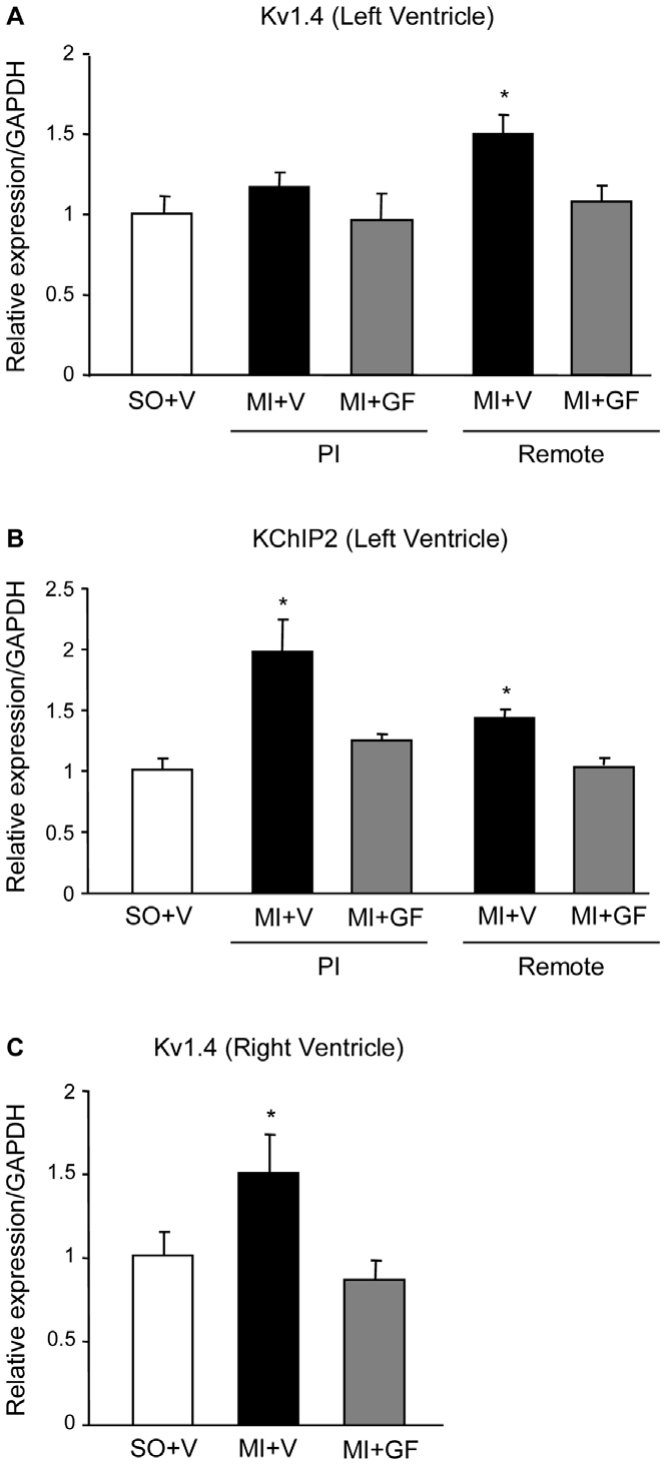
Expression of K channel isoforms. Quantification of Kv1.4 (A, C) and KChIP2 (B) m-RNA levels, in ventricular myocardium. PI: peri-infarcted; Remote: spared left ventricular myocardium. Each bar represents the mean of 4 different samples in triplicate. * p<0.05 vs. SO+V and MI+GF.

To determine whether an altered modulation of transient outward K current (I_to_) could affect ERP, the major isoforms of the rat alpha (Kv4.2, Kv4.3, Kv1.4) and beta (KChIP2) subunits were measured at mRNA level in the peri-infarcted and remote (left and right) ventricular myocardium of the MI+V and MI+GF rats, in comparison with SO+V. By RT-PCR analysis, the mRNA expression for Kv4.2 and Kv4.3 alpha subunits in each myocardial region was alike in all animals (data not shown). Conversely, Kv1.4 and the accessory subunit of the K channel KChIP2 were significantly increased by MI (MI+V group), in the remote left and right ventricular myocardium (Kv1.4: about +50%, p<0.05) (Fig. 4A, C), and peri-infarcted and remote LV myocardium (KChIP2: about +100% and +20%, p<0.05) (Fig. 4B). In both cases, the rise was prevented by cytokines (MI+GF group).

#### Connexin43 expression

**Figure 5:**
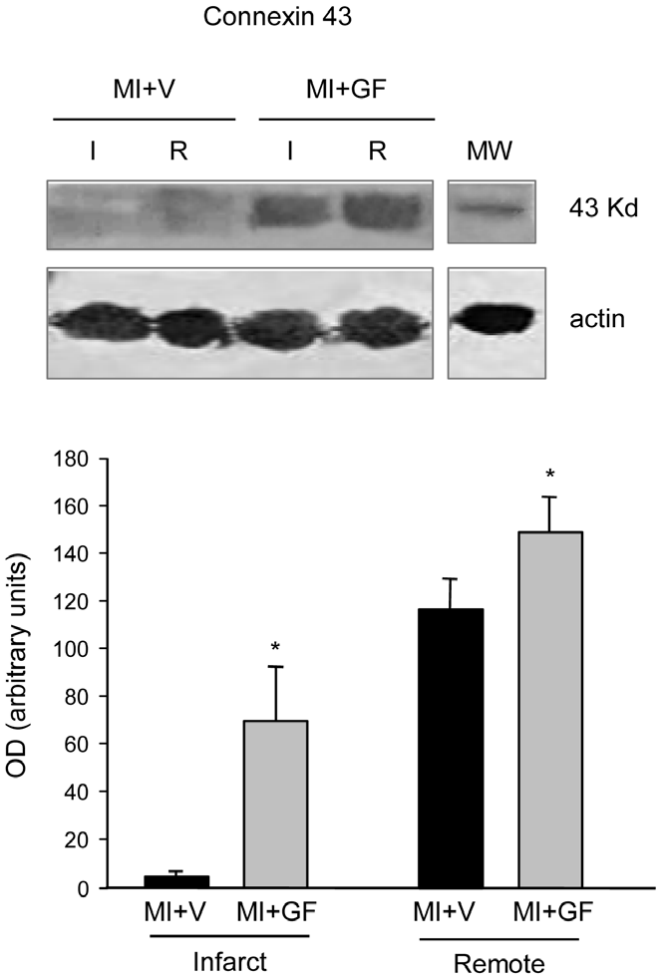
Quantification of connexin43 protein expression. Western blot analysis of connexin43 protein expression in infarcted (I) and remote (R) left ventricular myocardium of MI+V and MI+GF hearts. MW = molecular weight marker; OD = optical density. * p<0.05 vs. MI+V.

To confirm the positive effects on electrical function evoked by the intramyocardial injection of GFs, the Cx43 expression was evaluated in the infarcted and remote portions of the rat ventricles. The levels of Cx43 within the infarcted region were negligible in the MI+V group whereas local GF administration increased by about 10-fold (p<0.05) the expression of the gap-junctional protein (Fig. 5), suggesting a better electrical coupling within the scarred partially regenerated myocardium. Similar findings, although of lower magnitude (about 30% increase, p<0.05), were observed in the remote myocardium (Fig. 5).

### 2) Mechanical function

#### Echocardiographic and Hemodynamic Study

**Figure 6:**
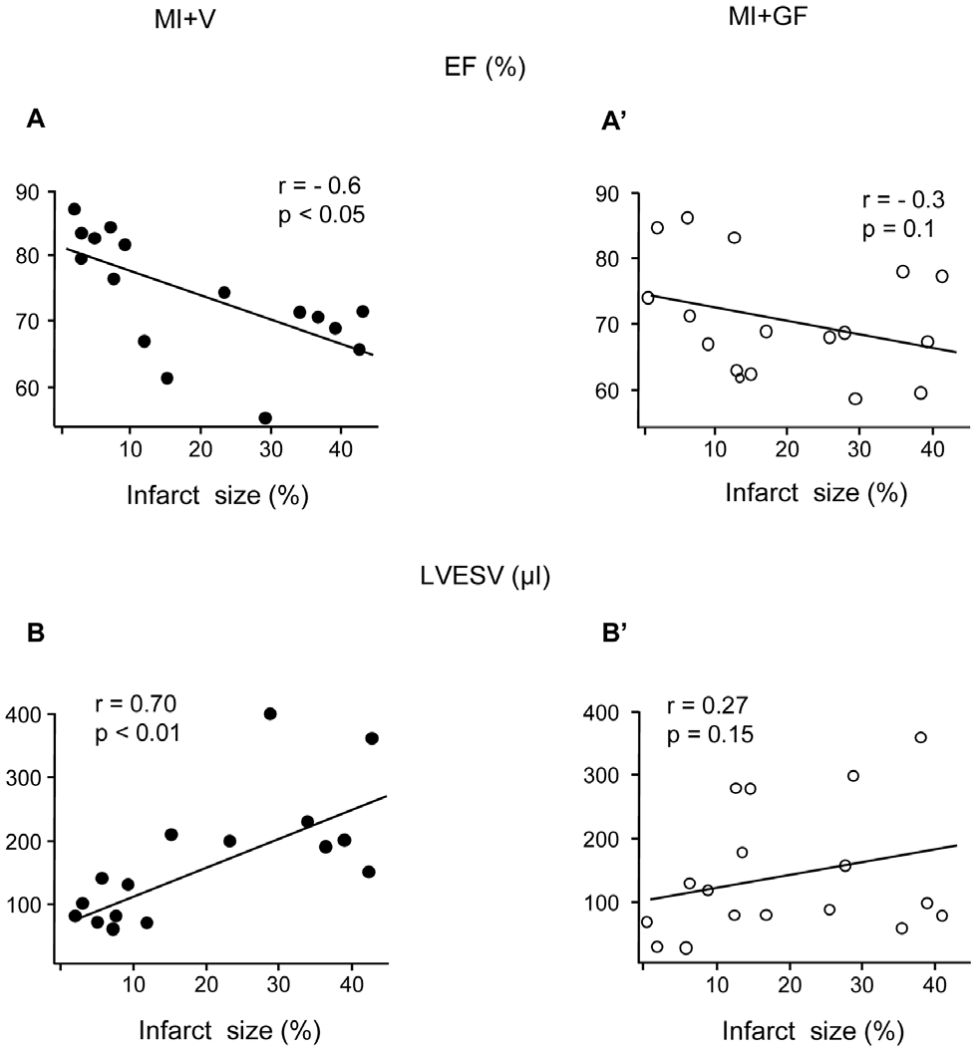
Blunting effect of GF treatment on the correlation between infarct size and echocardiographic parameters. Linear correlations between infarct size and ejection fraction (EF) and left ventricular end-systolic volume (LVESV), in MI+V (A–B) and MI+GF (A'–B') animals. The statistical significance of correlations was limited to the MI+V group.

Before GF/V injection, echocardiographic measurements revealed the expected global deterioration in cardiac function in the MI group when compared with the SO group (Table S2). Two weeks after injection, in the MI+V animals, the morpho-functional properties of the left ventricle either remained unchanged or underwent a slight further deterioration (Table S2). In contrast, in the MI+GF rats, the treatment was followed by an improvement in the values of left ventricular end-diastolic diameter (LVEDD) and left ventricular end-diastolic volume (LVEDV), which approached those measured in the SO+V group (Table S2). Moreover, in the MI+V group, infarct size (morphometrically determined, see below) was negatively correlated with fractional shortening and ejection fraction and positively correlated with left ventricular systolic diameter (LVSD) and left ventricular end-systolic volume (LVESV) (Fig. 6A–B). These correlations disappeared in MI+GF group (Fig. 6A'–B') further supporting the hypothesis that the administration of HGF+IGF-1 induced a partial recovery of ventricular mechanical performance.

**Figure 7:**
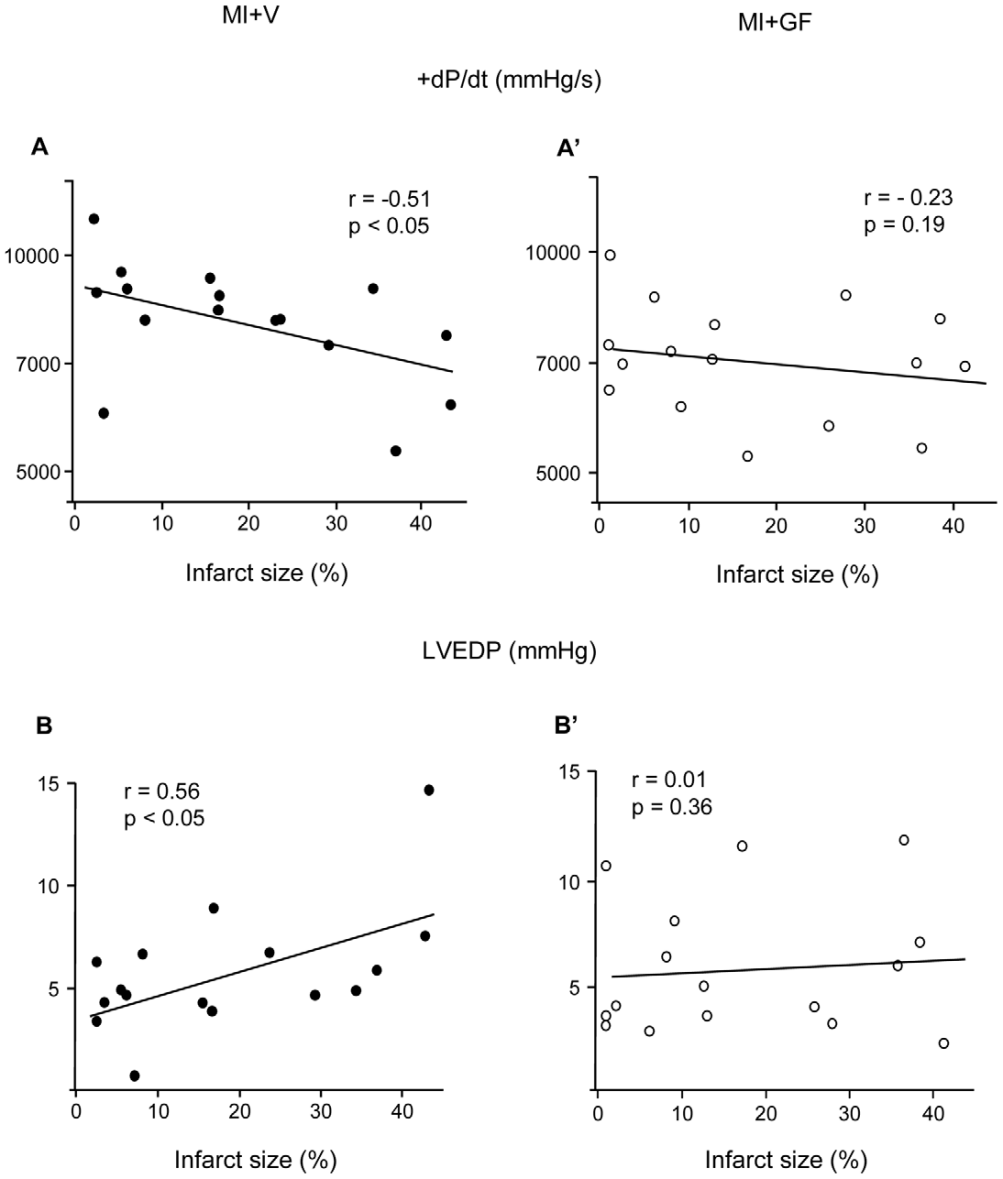
Blunting effect of GF treatment on the correlation between infarct size and hemodynamic parameters. Linear correlations between infarct size and maximum rate of ventricular pressure rise (+dP/dt) and left ventricular end-diastolic pressure (LVEDP), in MI+V (A–B) and MI+GF animals (A'–B'). The statistical significance of correlations was limited to the MI+V group.

In line with echocardiographic data, invasive hemodynamic measurements indicated that the impairment of dP/dt and left ventricular end-diastolic pressure (LVEDP) was correlated with post mortem determination of infarct size in the MI+V group (Fig. 7A–B) but not in the MI+GF group (Fig. 7A'–B'). These findings confirm that GF treatment abolishes the proportional deleterious effect of infarct size on the magnitude of LV mechanical performance.

### 3) Cardiac remodeling and regeneration

#### Cardiac Anatomy and Structure

We tested whether the beneficial effects of cytokine treatment on the electromechanical properties of the infarcted heart had a structural/anatomical counterpart.

Compared with the SO+V animals, the MI+V group exhibited a significant increase in chamber volume associated with a moderate thinning of the left ventricular wall, resulting in a decreased mass-to-chamber volume ratio (Table 1). These markers of unfavorable remodeling, representing the major anatomical determinants of heart failure, were attenuated in the MI+GF rats (Table 1).

**Table 1:** Left ventricular geometry and infarct size.

	SO+V (n = 15)	MI+V (n = 23)	MI+GF (n = 22)
LV mass (mm^3^)	769±43	1046±35 *	1031±17 *
LV wall thickness (mm)	2.2±0.1	2.0±0.1	2.2±0.1
LV chamber diameter (mm)	5.5±0.2	7.2±0.2 *	6.7±0.2 *
LV chamber volume (mm^3^)	199±11	413±25 *	336±18 * #
mass/chamber volume	4.1±0.1	2.4±0.1 *	2.9±0.2 *
Infarct size (%)		19.4±3.2	19.9±2.9

Mean values±SE of left ventricular (LV) geometrical properties measured in SO+V, MI+V and MI+GF groups.

*p<0.01 significant differences vs. SO+V group;

# p<0.05 significant differences vs. MI+V.

**Figure 8:**
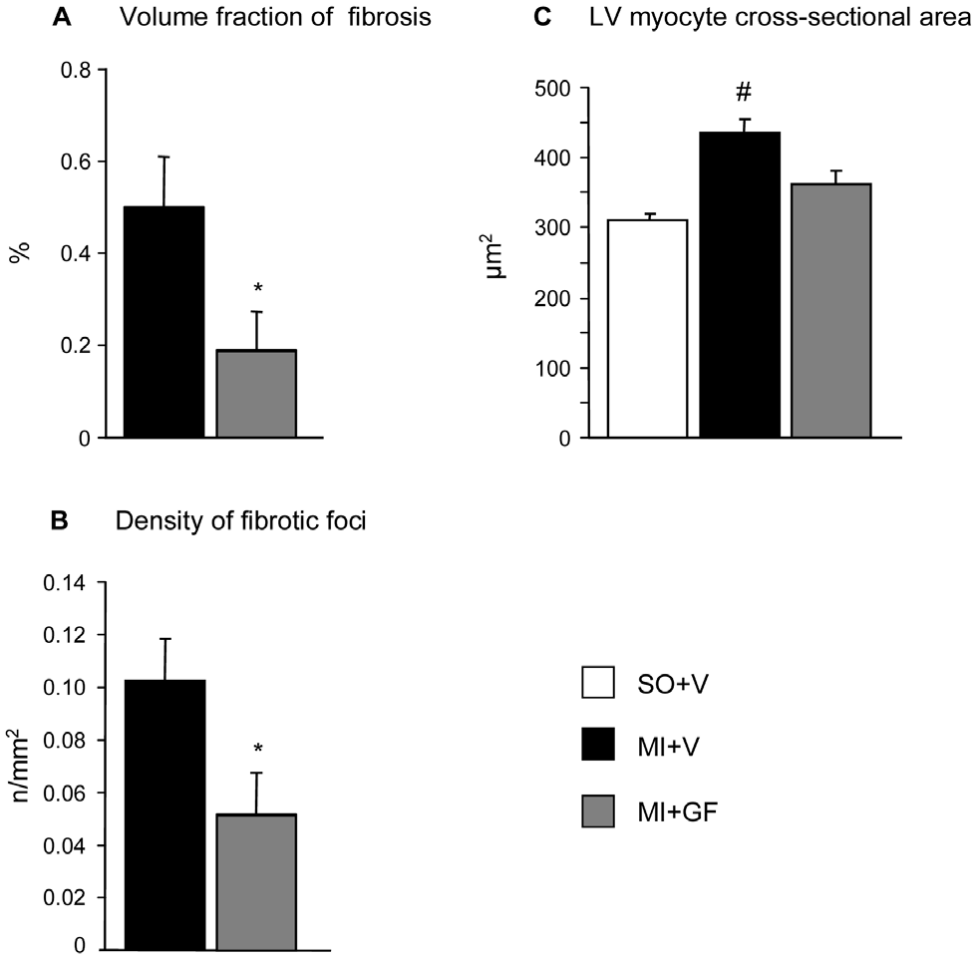
Myocardial fibrosis and left ventricular myocyte size. Mean values±SE of volume fraction of fibrosis (A) and numerical density of fibrotic foci (B), morphometrically evaluated in the remote left ventricular (LV) myocardium of MI+V and MI+GF hearts. In (C), mean values±SE of the LV myocyte cross-sectional area. * p<0.05 vs. MI+V; # p<0.05 vs. SO+V.

Myocardial fibrosis and small foci of collagen accumulation, uniformly distributed throughout the LV wall, were detected in the spared non-infarcted ventricular myocardium of both the MI+V and MI+GF groups. However, the volume fraction of interstitial fibrosis and the number of foci of replacement fibrosis were more than 2-fold lower in the treated MI+GF hearts (p<0.05; Fig. 8A–B).

The measurement in tissue sections of cross sectional area of transversally oriented spared myocytes showed that the average cell size was significantly increased only in the MI+V rats (p<0.05 vs. SO+V; Fig. 8C), indicating that reactive cellular hypertrophy was reduced by intramyocardial injection of GFs.

#### Immunohistochemical analysis of myocardial regeneration

**Figure 9:**
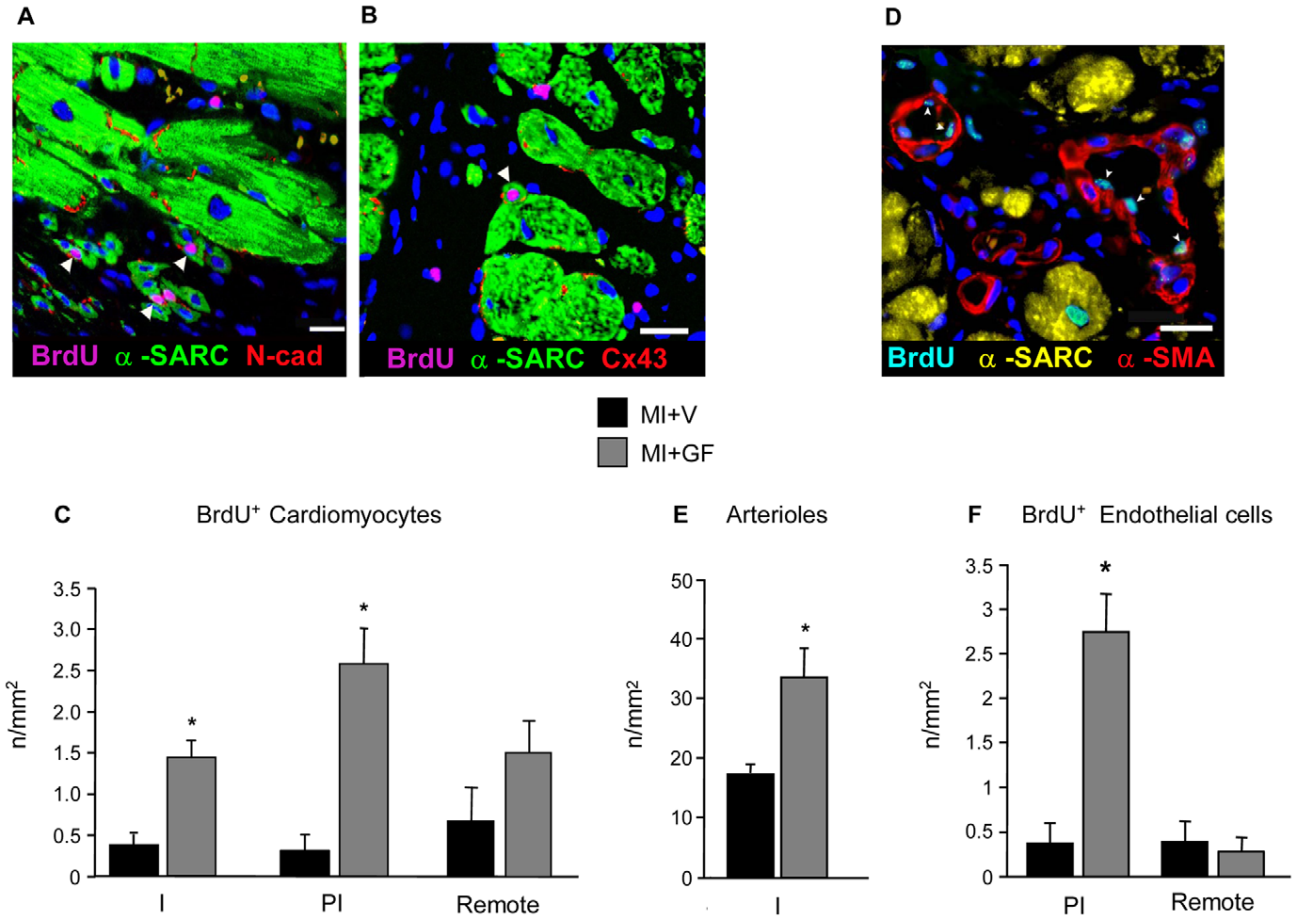
Myocyte and vessel regeneration. Immunofluorescence staining of sections of the infarcted myocardium of GF treated rats (A, B, D). Myocyte regeneration. (A–B): arrowheads indicate BrdU labeled (pink fluorescence) nuclei of small α-sarcomeric actin positive cardiomyocytes (α-SARC, green fluorescence) showing in red fluorescence mechanical (N-cad = Ncadherin) and electrical (Cx43 = connexin43) connections. Blue fluorescence corresponds to DAPI labeling of nuclei. Scale Bars: A) 20 µm; B) 50 µm. (C) Bar graphs illustrate the quantification of proliferating cardiomyocytes in the infarcted (I), peri-infarcted (PI) and remote myocardium of vehicle (V) and Growth Factor (GF) treated hearts. * p<0.05 vs. MI+V. Vessel regeneration. (D): Arrowheads point to BrdU+ (bright fluorescence) endothelial cells lining α-smooth muscle actin (α-SMA, red fluorescence) arterioles. Blue fluorescence corresponds to DAPI labeling of nuclei. Scale Bar: 50 µm. Bar graphs (E–F) illustrate the quantification of the absolute number of arterioles in the infarcted (I) myocardium (E), and of endothelial cells in the peri-infarcted (PI) and remote myocardium (F) of vehicle (V) and Growth Factor (GF) treated hearts. * p<0.05 vs. MI+V.

Areas of myocardial regeneration were detected in the infarcted portion of GF treated rat hearts. Small BrdU+ myocytes, expressing N-Cadherin (Fig. 9A) and Cx43 (Fig. 9B) were observed, indicating that newly formed, electromechanically coupled cardiac myocytes resulted from local injection of GFs. Within the two weeks from cytokine injection a nearly 3-fold increase in cycling myocytes accumulated in MI+GF infarcts when compared with the MI+V ones (Fig. 9C). New myocytes properly integrated with the surrounding tissue were also present in the peri-infarcted and remote myocardium of treated animals, suggesting that local delivery of GFs exerted a global beneficial effect on the heart (Fig. 9C). Although similar findings could occasionally be detected in untreated chronic infarcts, the quantitative estimation clearly indicated that the regenerative processes were markedly enhanced in the MI+GF group in both the peri-infarcted and infarcted areas (Fig. 9C). Importantly, the newly formed myocardium was properly perfused, as indicated by the significant increase in the density of arteriolar profiles (two-fold) and BrdU+ endothelial cells (five-fold) (Fig. 9D–F).

**Figure 10:**
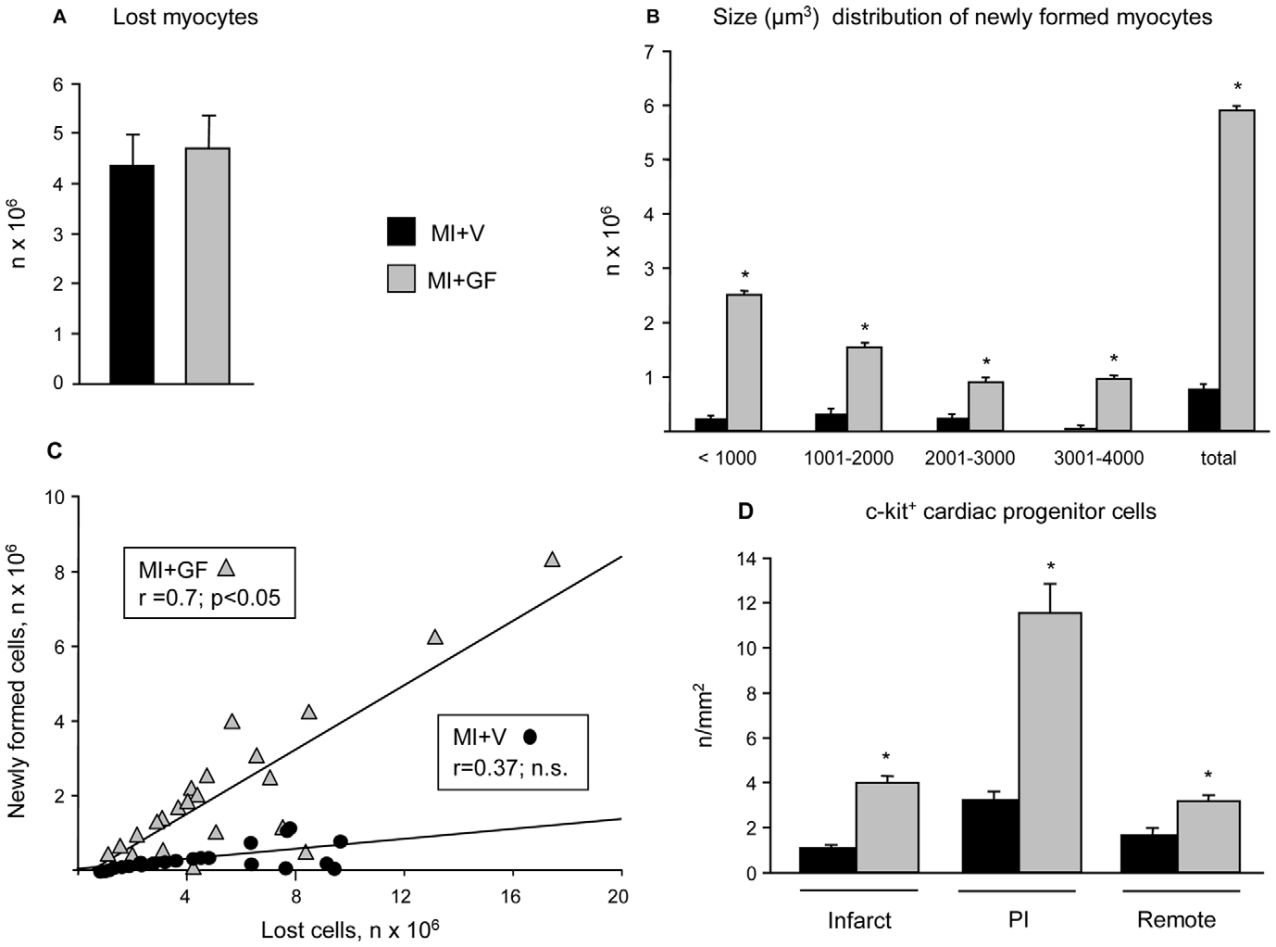
Quantitative analysis of cardiac progenitor cells and cardiomyocyte loss and regeneration. In the presence of similar amounts of myocyte loss (A), compared to vehicle treated infarcts (MI+V), intramyocardial injection of Growth Factors (GF) produced a significant number of small, immature and larger, more mature cardiomyocytes (B) whose generation was directly related to the number of lost cells (C). Treatment with GFs was also able to increase the incidence of c-kit+ cardiac progenitors in the infarcted, peri-infarcted (PI) and remote myocardium (D). * p<0.05 vs. MI+V.

Infarct size, as measured by the amount of myocytes lost 6 weeks after coronary artery ligation (4 weeks prior + 2 weeks after GF/V injection), was similar in the two experimental groups. As shown in Fig. 10A, nearly 4.5×10^6^ myocytes were lost in the MI+V and MI+GF hearts. Within the infarcted area, cytokine treatment promoted the formation of 15.6±2.5 mm^3^ of new myocardium in which an average of 5.9±0.1×10^6^ developing myocytes ranging in size from 200 to a maximum of 4000 µm^3^ were present (Fig. 10B). Most newly formed cardiomyocytes had a volume of less than 2000 µm^3^, although cytokine treatment was able to generate a consistent number of cells approaching a volume of 4000 µm^3^. This magnitude of myocardial regeneration resulted in a 21.2±1.7% replacement of the infarcted volume by newly formed myocardium. Conversely, myocardial repair in untreated infarcts was unremarkable. It is noteworthy that the generation of new myocytes increased linearly with the amount of cell loss in GF treated animals (Fig. 10C). A similar reparative capacity was not observed in untreated rats. On the other hand, myocardial regeneration was associated with intense activation and translocation of resident progenitor cells (Fig. 10D). In comparison with MI+V, a significant (p<0.05) increase in the incidence of c-kit+ cells was produced by GFs in the infarcted, peri-infarcted and remote myocardium. Altogether these findings strongly suggest that GF-induced cardiac repair was mediated by a multipotent stem cell population.

## Discussion

The induction or exacerbation of arrhythmias is a major concern in stem cell based therapies for cardiac repair, and the effect of regenerative treatments on cardiac electrogenesis is still under debate [5]–[15], [46]–[47]. The present results demonstrate that intra-myocardial injection of HGF and IGF-1 in a rat model of healed myocardial infarction caused a significant decline in arrhythmias of conscious freely moving animals subjected to stressful conditions. Concurrently, the effective refractory period (ERP) at the ventricular surface of GF treated hearts was prolonged without changes in dispersion of refractoriness, QTc interval duration and mRNA levels of potassium channel α-subunits Kv4.2 and Kv4.3. In contrast, lower mRNA levels of α-subunit Kv1.4 and β-subunit KChIP2 were found in the remote and remote/peri-infarcted myocardium respectively.

In support of previous data [26]–[28], we also found that resident CPCs, stimulated locally by GF treatment, invaded the scarred myocardium and generated new electromechanically-connected myocytes and vessels. Additionally, experimental evidence was provided for a partial recovery of mechanical competence in the regenerated heart, associated with attenuation of unfavorable remodeling. Finally, newly formed myocardial structures together with a reduction in reactive cellular hypertrophy and interstitial fibrosis were detected in peri-infarcted and remote myocardium, suggesting that local delivery of GFs also exerted beneficial effects in the spared tissue.

### Recovery of Electrical Function

Experimental electrophysiological studies aimed at assessing the proarrhythmic or antiarrhythmic potential of stem cell based therapies in vivo have been carried out mostly in anesthetized animals and in a few cases in conscious animals in baseline conditions [44]. While anesthesia can have confusing effects, the incidence of arrhythmias in baseline conditions is generally low. To circumvent these limitations, we measured the proneness to arrhythmias in conscious freely moving rats subjected to the resident-intruder test [32] which, by mimicking a natural situation, ensured that the mechanisms of arrhythmias acting during stress were in all probability the same which may trigger arrhythmias in everyday life of social animals. The procedure is known to consistently induce an intense activation of the autonomic nervous system, with a shift of the sympatho-vagal balance towards a sympathetic predominance, resulting in arrhythmias even in normal animals [45]. Stress-induced ventricular arrhythmias were reduced by about a half following GF injection, while no changes were observed in untreated rats, indicating a protective effect of local delivery of cytokines.

It has been recently reported [6] that important issues to consider when assessing the effect of stem cell based myocardial regeneration on cardiac electrical competence include: (i) intrinsic electrophysiological properties of stem cells, (ii) modulated graft-host and/or graft-graft electromechanical coupling, (iii) changes in ion channel function, (iv) induced heterogeneity, and (v) altered myocardial tissue architecture comprising heterogeneous sympathetic nerve sprouting. All these factors may affect cardiac electrogenesis either by acting alone or in concert with the arrhythmic substrate of the injured myocardium.

We found that dispersion of refractoriness was similar in treated and untreated animals, as were the indirect indexes of autonomic input to the heart (SD_RR_ and r-MSSD), signifying that GFs did not alleviate arrhythmia susceptibility by affecting repolarization heterogeneity [46] or cardiac sympathetic activity [47].

Quantitative RT-PCR analysis of the genes encoding the Ca2+-independent transient outward potassium current (I_to_) revealed that the transcript expression of Kv1.4 was up-regulated in the remote left ventricle only in untreated rats. Kv1.4 is the main channel subunit contributing to I_to_ during the embryonic development of mice and rats. After birth, Kv1.4 expression levels decrease, while Kv4.2 and Kv4.3 are up-regulated [48]. Importantly, cardiac hypertrophy induces a re-expression of Kv1.4 mRNA [49]. Furthermore, the rise in mRNA levels of potassium channel β-subunit KChIP2 produced by myocardial infarction was cancelled out by GF administration. Recent studies documented higher mRNA levels of KChIP2 in cardiac hypertrophy [50] although a full appreciation of this result is impossible, since to date the role of KChip(s) subunit(s) has not been clearly elucidated. In conclusion, the lower mRNA levels of Kv1.4 and KChIP2 subunits produced by GF injection might be associated with the reduced reactive cellular hypertrophy following cardiac repair. These findings, together with cardiac anatomical and morphometric data, indicate that GF treatment may succeed in partially controlling endogenous arrhythmic substrates by limiting myocardial damage, and reducing myocardial fibrosis and reactive cellular hypertrophy in the remote myocardium, thereby helping to prevent the occurrence of reentry circuits.

By using epicardial multiple-lead recording, we showed that cytokine injection resulted in a marked prolongation of ERP, while conduction velocity (CV) was unchanged. The combined effect of CV and ERP on impulse propagation is defined by the wavelength L = CV x ERP, implying that a longer L (by increasing CV and/or ERP) will reduce the likelihood that a single or multiple reentrant circuits can be accommodated by the heart [43]. Hence, ERP changes observed in GF treated rats could reduce the ability of cardiac tissue to elicit an abnormal propagated response by increasing L, thus hampering reentry. The finding that epicardial QTc values were similar in all animals indicates that mechanisms other than lengthening of action potential duration (APD) were responsible for the longer ERP in GF treated rats. Accordingly, either membrane currents affecting APD were not influenced by GFs or, if they were, the net modification might not be large enough to manifest itself at the tissue level. Indeed, our data show that mRNA levels of the potassium channel α-subunits Kv4.2 and Kv4.3 did not exhibit any significant difference between untreated and HGF+IGF-1 treated hearts, suggesting that the transient outward potassium current (I_to_), which contributes conspicuously to determine APD, was unaltered.

On the other hand, recent studies have demonstrated that IGF-1, alone or in combination with other growth factors, increases Cx43 expression in cardiac myocytes [10]. In support of these findings, we showed that GF administration significantly increased the expression of the gap-junctional protein Cx43, providing evidence for an increased electrical coupling within the scarred, partially regenerated myocardium as well as in the remote spared tissue. A better intercellular electrical connection is considered as being an important antiarrhythmic factor [10]–[15], [51] and could also result in ERP prolongation. It has recently been remarked [52] that the amount of current needed for cardiac excitation by point stimulation is modulated by the properties of electrical coupling of the interconnected myocytes via gap-junctions. The smallest myocardial region capable of initiating a propagated action potential (“liminal length”) is inversely related to the extra- and intracellular resistivities, which are largely dependent on interstitial fibrosis and electrical coupling between myocytes respectively [52]–[53].

The role of myocardial geometry in the charge threshold for excitation was documented by computing the S-D curve, which shifted to the right when the “liminal length” increased (i.e. reduced excitability) and to the left when it decreased (i.e. increased excitability) [53]. Changes in “liminal length” resulting from different degrees of electrical coupling between myocytes are also expected to affect cardiac refractoriness, which will be increased by an increase in “liminal length” and reduced by its reduction. Concurrently, the longer ERP and the associated tendential shift to the right of the S-D curve induced by HGF+IGF-1 administration might be attributed to the higher expression of Cx43 and the longer “liminal length” resulting from an enhanced cardiomyocyte interconnection.

### Cardiac Regeneration, ventricular remodeling and recovery of mechanical function

Recent studies in animal models of chronic myocardial infarction [28] have documented a negative impact of the scarred myocardium on the migration and engraftment of CPCs when compared with acute infarcts. Yet, in spite of the less favorable environment related to chronic collagen deposition after infarction, GF-activated CPCs retain the ability to infiltrate the scar, digest part of the connective tissue, and form cardiomyocytes and coronary vessels via enhanced activity of metalloproteases. In support of these findings [28], we showed that HGF and IGF-1 administration triggered the proliferation and mobilization of CPCs, leading to newly formed functionally competent myocardial tissue. Importantly, proliferating myocytes expressed N-Cadherin and Cx43 and linearly increased with the amount of cell loss. Coronary arterioles and capillary structures were concurrently produced with myocytes, tending to preserve blood supply and oxygen diffusion to the surrounding cells. Ultimately, GF-induced activation of CPCs elicited a sustained regenerative response that resulted in a 20% rescue of the infarct by replacing fibrotic tissue with viable myocardium.

GF treatment attenuated ventricular dilation, increased ventricular mass/chamber volume ratio, and reduced collagen accumulation in the spared myocardium more than 2-fold while restraining the hypertrophic response of myocytes to the segmental loss of cells.

As shown by the echocardiography findings, post-MI ventricular dysfunction tended to recover in GF treated infarcts. Furthermore, echocardiographic and hemodynamic data documented that the significant, negative correlation of cardiac mechanical function with the extension of the infarcted area in untreated animals was lost as a result of intramyocardial injection of GFs.

It could be argued that a single injection of cells or GFs hardly promotes a sustained and prolonged cardiac repair in the absence of a robust activation of autocrine/paracrine processes. Moreover, the question remains as to whether a 20% recovery of myocardial mass within the infarct, mostly consisting of small contractile cells, is sufficient to prevent the evolution of myocardial infarction toward cardiac decompensation. However, infarct size is proportional to the number of lost cardiomyocytes, representing the major determinant of unfavorable LV remodeling and its chronic evolution toward heart failure [40]. Hence it is of relevance that the GF-mediated generation of young functionally competent cardiomyocytes succeeds in making cardiac function and anatomy independent of the amount of tissue lost by the occlusion of the supplying coronary artery. These results suggest that locally delivered GFs, by reversing the hostile microenvironment of scarred myocardium, promote a cardiac repair which is proportional to tissue demand. Intriguingly, this phenomenon has been observed in clinical experience of cell therapy for ischemic cardiomyopathy [54]–[56], documenting greater improvement in global cardiac function in patients who had a lower ejection fraction at baseline. Thus, regenerative approaches seem to exert a more beneficial effect on larger myocardial infarcts.

### Conclusion

Altogether, this study indicates that local injection in scarred infarcts of active peptides able to recapitulate an endogenous cell program aimed at generating functionally competent myocardium, besides having a positive impact on the mechanical properties of the injured heart, results in a significant decline in proneness to arrhythmias. Both ERP prolongation (likely mediated by improved intercellular electrical coupling leading to increased liminal length for impulse formation) and positive ventricular structural remodeling contribute to the reduced ventricular arrhythmogenesis.

## Supporting Information

File S1 Detailed description of the experimental design, surgical procedures, electrophysiological measurements (telemetry ECGs and multiple-lead epicardial recording), echocardiographic and hemodynamic measurements, morphometrical and immunohistochemical analyses, and biochemical and molecular biology analyses (electrophoresis and immunoblot assay, and RT-PCR).

Table S1 Outline of the experimental protocols.
(DOC)

Table S2 Echocardiographic data.
(DOC)

Figure S1 **Epicardial multiple-lead recording: electrode arrays and procedures.** (A) Electrode array positioned on the anterior aspect of the ventricular surface. (B) Schematic representation of the 5×5 (in red) and 8×8 electrode arrays showing the positions (larger circles) of the 5 and 8 selected electrodes used for specific pacing protocols. (C–D): Unipolar electrograms collected by means of the 8×8 electrode array during normal sinus rhythm (C) and ventricular pacing at the electrode indicated by the pulse symbol (D). In (E), example of paced activation isochrone map used for computing conduction velocity longitudinally (blue-arrow) and transversally (red-arrow) to fiber orientation; numbers on each isochrone line indicate the activation time in ms. In (F), strength-duration curve obtained in a control rat by plotting pulse threshold current I as a function of pulse duration T (Rh: Rheobase, Chr: Chronaxie).
(TIF)
