# Supplementary material for: Growth Factor-Induced Mobilization of Cardiac Progenitor Cells Reduces the Risk of Arrhythmias, in a Rat Model of Chronic Myocardial Infarction
Source: PLoS One. 2011 Mar 18;6(3):e17750. doi: 10.1371/journal.pone.0017750 (PMC3060871; doi:10.1371/journal.pone.0017750)
Supplement: File S1 — Detailed description of the experimental design, surgical procedures, electrophysiological measurements (telemetry ECGs and multiple-lead epicardial recording), echocardiographic and hemodynamic measurements, morphometrical and immunohistochemical analyses, and biochemical and molecular biology analyses (electrophoresis and immunoblot assay, and RT-PCR). [file pone.0017750.s001.doc]

**SUPPORTING INFORMATION**

Growth Factor-Induced Mobilization of Cardiac Progenitor Cells Reduces the Risk of Arrhythmias, in a Rat Model of Chronic Myocardial Infarction

Bocchi L, Savi M, Graiani G, Rossi S, Agnetti A, Stillitano F, Lagrasta C, Baruffi S, Berni R, Frati C, Vassalle M, Squarcia U, Cerbai E, Macchi E, Stilli D, Quaini F, Musso E

**Corresponding author**:

Ezio Musso

Dipartimento di Biologia Evolutiva e Funzionale, Università di Parma, Parma, Italy

E-mail: ezio.musso@unipr.it

**Methods**

**Animals and housing**

The study population consisted of 207 male Wistar rats (*Rattus norvegicus*) bred in our departmental animal facility, aged 12-14 wk, weighing 300-350 g. The animals were kept in single-sex groups of four individuals from weaning (4wk after birth) until the onset of the experiments, in a temperature-controlled room at 20–24°C, with the light on between 7.00 AM and 7.00 PM. The bedding of the cages consisted of wood shavings, and food and water were freely available.

The animals were **subjected** either **to myocardial infarction (MI group) or sham operation (SO group). Four weeks later, all animals were treated with growth factors (GFs) or vehicle (V) and** assigned to the following groups: **MI+GF, MI+V and SO+V. GFs were** Hepatocyte Growth Factor (HGF) and Insulin-Like Growth Factor-1 (IGF-1).

**Fifty four rats died in the peri-operative periods and 20 additional rats, assigned to the MI+GF or MI+V group, were excluded from the study because infarction could not be clearly detected at autopsy, leaving a total of 133 rats. Owing to the multifactorial nature of arrhythmogenesis in myocardial infarction**, all animals with anatomically documented MI were used in the study, even if infarct size was small.

**Outline of the experimental protocols**

**The experimental design and measurements performed in the study are summarized in Table S1.**

**Sixty rats were chronically instrumented with a Telemetry-ECG system (TE rats, Table S1). After one week, a 30-minute continuous baseline ECG was recorded to rule out the presence of spontaneous arrhythmias. The animals were then subjected either to myocardial infarction (MI group, 45 rats) or to sham operation (SO group, 15 rats) and, four weeks later, investigated for vulnerability to arrhythmias and cardiac mechanical performance by (i) telemetry-ECG recording in baseline condition and stress-induced autonomic stimulation (social stress procedure) and (ii) echocardiograpy. Afterwards, all rats were re-operated on and intra-myocardial injections of growth factors or vehicle were performed (MI+GF group, 22 rats; MI+V, 23 rats; SO+V, 15 rats). Simultaneously** with GF or V (GF/V) administration, **selected subgroups of MI+GF (n=14) and MI+V rats (n=11) were also treated with** 5-Bromo-2’-deoxycytidine **(BrdC),** to evaluate the cumulative amount of cell proliferation. **Two weeks later, vulnerability to arrhythmias and cardiac mechanical function were re-determined by a new series of telemetry-ECG and echocardiographic measurements and, subsequently, hemodynamic data were invasively collected. Finally, at sacrifice, the heart was perfusion fixed for anatomical studies (all TE rats), and morphometric and immunohistochemical studies (selected subgroups of animals).**

**In 43 additional rats, assigned to the MI+GF (n=24) or MI+V (n=19) groups, further electrophysiological investigations were performed by Epicardial Multiple-lead recording (EM rats, Table S1). In all animals, four weeks after MI, 25-64 unipolar electrograms (EGs) were collected from as many sites of the infarcted epicardial surface and surrounding areas. EGs were recorded during sinus rhythm or specific pacing protocols for determining basic cardiac electrophysiological properties (conduction velocity, excitability and refractoriness) as well as dispersion of refractoriness and QT interval duration. Subsequently, growth factors or vehicle were administered to the proper subgroups. Two weeks later, the animals were re-operated on, all electrophysiological measurements were repeated and, at sacrifice, the heart was perfusion fixed for identifying the** infarcted portion of the ventricle.

**Finally, in 30 rats assigned to the MI+GF, MI+V and SO+V groups, two weeks after GF/V injection the heart was excised and frozen for Molecular Biology analyses (MB rats, Table S1) which included electrophoretic and immunoblot assays (5 MI+GF and 5 MI+V animals) and** quantitative RT-PCR measurements **(9 MI+GF, 8 MI+V and 3 SO+V animals).**

**The details of the procedures employed in the study are described below.**

**Chronic instrumentation for telemetry-ECG recording**

The animals were chronically instrumented with a miniaturized transmitter for telemetry-ECG recording (model TA11CTA-F40, Data Sciences, St Paul, MN, USA), as previously published [1]. Briefly, each rat was anesthetized with ketamine chloride 40 mg/kg ip (Imalgene, Merial, Milano, Italy), plus medetomidine hydrochloride 0.15 mg/kg ip (Domitor, Pfizer Italia S.r.l., Latina, Italy). A ventral celiotomy was performed and the body of the transmitter (25x15x8 mm) was placed in the abdominal cavity. One recording lead was fixed to the dorsal aspect of the xiphoid process, close to the apex of the heart. The other lead was subcutaneously tunneled on the thorax toward the upper insertion of the sternohyoid muscle. Here, the wire was formed into a U shape, pushed under the muscle and then along the trachea into the anterior mediastinum, to get the tip of the recording lead close to the right atrium. Finally, the muscle and skin layers were separately sutured.

After surgery, all animals were given atipamezole hydrochloride 0.15 mg/kg im (Antisedan, Pfizer Italia S.r.l.), flunixin 5 mg/kg im (Finadyne, Schering-Plough S.p.a, Milano, Italy), gentamicine sulphate 10 mg/kg im (Aagent, Fatro, Milano, Italy), and kept warm with infrared lamp radiation. Antibiotic therapy was continued in the following three days while the animals were individually housed.

**Social stress procedure and telemetry-ECG data acquisition and processing**

Social stress was obtained by introducing the experimental animal (intruder) into the territory of an unfamiliar conspecific male (resident) belonging to an aggressive wild strain of rats (Rattus norvegicus, Wild Type Groningen, WTG) (resident-intruder test paradigm) [2]. High levels of aggression by the resident animal were achieved and maintained by cohabitation with a female and a training procedure accomplished in the two weeks preceding the intruder test.

All experimental sessions were carried out during the light phase, between 9.00 AM and 1.00 PM. Each session consisted of two successive 15-minute telemetry-ECG recordings which were performed while the experimental animal was respectively: (i) left alone and undisturbed in its home cage (baseline conditions) and (ii) exposed to the social stress procedure (test period). During the entire recording session, a telemetry-ECG receiver (model CTR85-SA, Data Sciences) was placed under the experimental cage. ECGs, provided as analog signals at the output of the receiver, were continuously monitored on an oscilloscope and simultaneously routed to a personal computer via an analog-to-digital conversion board (12 bits, 1000 Hz sampling rate). A real-time acquisition program allowed the digital data to be continuously recorded and stored on computer memory for off-line processing.

The data were analyzed by using a custom made software package for measuring R-R interval, interactively detecting ventricular rhythm disturbances and computing time domain indexes of heart rate variability, as indirect measurements of the autonomic input to the heart. Ventricular arrhythmic events (VAEs) were classified as follows: (i) PVBs: isolated premature ventricular beats, and (ii) salvos: episodes of two or three consecutive PVBs [3]. The indexes of heart rate variability included: (i) the standard deviation of average R-R interval (SD_RR_) and (ii) the root mean square of successive R-R interval square differences (r-MSSD), which quantify the state of the balance between sympathetic and parasympathetic activities on the heart and the influence on heart rate of the parasympathetic branch respectively [4].

**Myocardial infarction**

Rats under ketamine-medetomidine anesthesia were intubated with a properly modified 16-gauge needle and ventilated (Rodent ventilator UB 7025, Ugo Basile, Comerio, Italy). With the aid of a dissecting microscope, a thoracotomy via the third-left intercostal space was performed and a 5-0 silk suture was passed with a tapered needle under the left anterior descending coronary artery, 2-3 mm from the edge of the left auricle, and tied securely. The development of a pale color in the distal myocardium on ligature, as noted by visual inspection, was taken as an index of the successful performance of coronary occlusion. The chest was then closed in layers, and a small catheter was introduced in the thorax to evacuate air and fluids. The rats were given atipamezole hydrochloride, removed from the ventilator, kept warm with infrared lamp radiation and eventually individually housed. Antibiotic therapy was given during the subsequent 3 days, as indicated above. Sham-operated rats were treated similarly, except that the ligature around the coronary artery was not tied.

**Intramyocardial injection of HGF+IGF-1 and BrdC delivery**

Four weeks after coronary ligature, left lateral thoracotomy was repeated in all animals under anesthesia and mechanical ventilation as described above. In MI+GF rats six intramyocardial injections were done with increasing concentrations of HGF (PeproTech EC, London, UK) and constant concentration of IGF-1 (PeproTech EC), diluted in sterile Phosphate Buffer Saline (PBS) [5]. The first injection was located near the atria, the second between the atria and the infarct and the last four at opposite sites of the border zone. In the MI+V and SO+V rats, corresponding regions of the heart were injected with vehicle. The concentrations of HGF were as follows: atria 50 ng/mL; atria-border zone 100 ng/mL; opposite sites of border zone 200 ng/mL. The concentration of IGF-1 was 200 ng/mL at all six injection sites. In the MI+GF rats, Rhodamine microspheres, 2.0 μm in diameter (FluoSpheres, Molecular Probes, Eugene, OR, USA), were added to the solution (v/v 5%) to ensure the successful injection. The volume of each injection was 10 μL.

In animals selected for BrdC administration, simultaneously with GF/V injection, an osmotic pump (model 2ML4, ALZET, Charles River Laboratories Italia S.p.A., Calco, Italy) was implanted subcutaneously in the inter-scapular region for continuous infusion of BrdC 0.6 mol/L (MP Biomedicals Europe, Illkirch, France) with a delivery rate of 2.5 μL/h. Infusion was maintained until the animals were sacrificed (two weeks). The long infusion time prompted us to use BrdC rather than BrdU, usually employed for the detection of DNA synthesis, because of the nearly six-fold higher solubility of BrdC, which is in vivo metabolically converted to BrdU.

**Echocardiographic measurements**

Serial echocardiograms were obtained in rats under ketamine-medetomidine anesthesia, using a Vivid 7 echocardiography machine (GE Healthcare Italia, Milano, Italy) equipped with 10-7 MHz phased array transducers. The anterior chest was shaved and the animals were placed in the left lateral decubitus position. Two-dimensional (2-D) and M-mode images were recorded from modified parasternal long axis and parasternal short axis views**.**

Diastolic and systolic anatomical and functional parameters were obtained from M-mode tracings at the mid-papillary level. The measurements included: (i) end-diastolic and systolic left ventricular diameter (LVEDD and LVSD respectively); (ii) left ventricular end-diastolic and end-systolic volume (LVEDV and LVESV) and (iii) ejection fraction and fractional shortening (EF, FS).

**Hemodynamic study**

The rats were anesthetized with droperidol+fentanyl citrate 1.5 mg/kg im (Leptofen, PHARMACIA & UPJOHN, Milan, Italy), which we have found to induce negligible changes in hemodynamic parameters as measured in conscious animals by means of telemetry recording (model PA-C40, Data Sciences) (data not shown).

The right carotid artery was cannulated with a microtip pressure transducer catheter (Millar SPC-320, Millar Instruments, Houston, TX, USA) connected to a recording system (Power Laboratory ML 845/4 channels, 2Biological Instruments, Besozzo, Italy) and systolic and diastolic blood pressure were determined. The catheter was then advanced into the left ventricle to measure: (i) left ventricular systolic pressure (LVSP) and end-diastolic pressure (LVEDP) and (ii) maximum rate of ventricular pressure rise (+dP/dt) and reduction (-dP/dt) in the closed-chest preparation.

**Epicardial multiple-lead recording**

EM rats under ketamine-medetomidine anesthesia and artificially ventilated were subjected to left thoracotomy. Body temperature was held constant at 37°C by infrared lamp radiation. A 5x5 or 8x8 row and column electrode array, fabricated on a surgical gauze patch, with 0.6 mm resolution square mesh, was positioned over the infarcted region and the surrounding areas with the upper border of the array just below the site of coronary ligature (**Fig. S1**A). The construction of the electrode array and the multiple-lead recording system have been described in detail before [6].

In each experiment, 25-64 unipolar EGs were recorded during normal sinus rhythm or specific pacing protocols at 5-8 selected electrodes of the array (**Fig. S1**B-D) by near-threshold, ≤1 ms cathodal current pulses, at a frequency slightly higher than spontaneous sinus rhythm (≈ 300 bpm). During pacing, unipolar EGs were recorded from all the electrodes except at the paced site (**Fig. S1**D). To minimize the invasiveness of the surgical procedure and ensure animal survival, all pre-GF/V-injection measurements were obtained using the 5x5 electrode array (**Fig. S1**B). Instead, the post-GF/V-injection measurements, carried out before sacrifice, were performed using the 8x8 array (**Fig. S1B**) to allow for a wider exploration of the epicardial surface. Furthermore, the appropriate positioning on the ventricular epicardium of the array was in some cases prevented by an unfavorable spatial relationship between heart and lungs in the open chest preparation. Because of this and the presence of scattered fibrotic areas in the infarcted region, only a fraction of the 25-64 sites explored by epicardial electrodes were excitable and/or enabled the recording of reliable EG signals.

The following measurements were carried out:

*Conduction velocity.* Activation times were estimated using the instant of the minimum time derivative of unipolar EGs during QRS, and were referenced to QRS onset or stimulus onset. From the activation times of paced beats an activation sequence (isochrone map) was determined where Conduction Velocity (CV) was computed longitudinally (CV-l) and transversally (CV-t) to fiber orientation (**Fig. S1**E). Specifically, CV-l was evaluated from electrodes distant from the pacing site on the long axis of the elliptical wavefront. CV-t was evaluated from electrodes on a line perpendicular to the long axis of the elliptical wavefront. It is known that the long axis of the elliptical wavefront is parallel to the local fiber direction at the pacing site [7].

*Excitability.* The 5-8 selected electrodes of the array (**Fig. S1B**) were initially paced with current pulses of decreasing strength (starting from liminar values) at a constant width of 5 ms till activation failed, thus identifying the threshold current. The procedure was repeated with current pulses of progressively shorter width, down to 0.01 ms.

The threshold currents as a function of pulse duration defined the Strength-Duration (S-D) curve (**Fig. S1F**) which is represented by the hyperbolic equation [8] relating threshold current strength I and pulse duration T:

I=Rh(1+Chr/T)

where Rh is the rheobase (i.e. the lowest intensity with infinite pulse duration which succeeds in eliciting a propagated response in excitable tissues) and Chr the chronaxie (i.e. the pulse duration having a threshold twice that of Rh) (**Fig. S1**F). The two parameters which univocally identify the S-D curve characterize tissue excitability.

*Refractoriness.* At each of the 5-8 selected electrodes of the array (**Fig. S1**B), ten baseline stimuli (S1), 1 ms width and twice diastolic threshold intensity, were delivered. The S1 pacing sequence was followed by an extra-stimulus (S2) whose delay from previous S1 was first progressively decremented by 10 ms steps until capture was lost and then progressively incremented by 2 ms steps till capture was resumed. The longest S1-S2 coupling interval that did not originate ventricular capture identified the effective refractory period (ERP) at a given electrode site, enabling the characterization of spatial differences in tissue refractoriness.

*Dispersion of refractoriness.* The degree of dispersion was evaluated by computing the ERP dispersion value taken as basic indicator of propensity to re-entrant arrhythmias [9]. In an individual animal, ERP dispersion was expressed by **the standard deviation of the mean ERP at all test sites [10].**

*QT interval duration.* In each animal, the duration of QT and corrected QT interval (normalized to cycle length: QTc) [11] was measured from the root mean square (RMS) signal computed from all of the 5x5 or 8x8 EGs and represented the average of 4 randomly selected beats. The RMS provides a composite measure of all electrical activity as a single positive-magnitude average of signals obtained from multiple unipolar EGs simultaneously [12], greatly improving the signal to noise ratio. QT duration was computed as the interval between QRS onset and the time of the minimum of the first derivative of RMS signal, during the descending limb of T-wave [13].

**Cardiac anatomy**

In all TE and EM rats, the abdominal aorta was cannulated, the heart was arrested in diastole with injection of CdCl_2_ solution (100 mmol/L, iv), and the myocardium was perfused in retrograde with 10% buffered formalin solution. The left ventricular chamber was filled with fixative at a pressure equal to the in vivo measured systolic pressure. The heart was then excised, placed in formalin solution (10%) for 24 hours and eventually tested for the presence of MI.

In the TE rats, the right ventricle (RV) and the left ventricle (LV) inclusive of the septum were separately weighed and the volume of the left ventricular myocardium was computed by dividing LV weight by the specific weight of the tissue (1.06 g/mL). LV chamber length was measured from the apex to the aortic valve. Three 1-mm thick transverse slices were cut from the base, mid-region and apex of the LV. The mid-slice was used to morphometrically compute LV wall thickness and chamber equatorial diameter (Image Pro-plus, Media Cybernetics, Bethesda, MD, USA, version 7.0). The LV chamber volume was calculated according to the Dodge equation, **which equalizes the ventricular cavity to an ellipsoid [**14]. The three slices were then embedded in paraffin and from each slice five-micrometer thick sections were cut and used for morphometric and immunohistochemical analyses (selected subgroups).

**Morphometric analysis**

*Infarct Size****.*** The infarcted portion of the ventricle was easily identifiable grossly and histologically. In the MI animals, the area of the damaged tissue from the endocardial to the epicardial layer and the total area of the viable myocardium were measured in the three slices corresponding to the base, mid-region and apex, indicated above. Because of shrinkage of the infarcted region and ongoing myocyte growth and death within the viable myocardium, the computation of the number of lost and remaining myocytes after infarction provides a more appropriate characterization of infarct size and extent of recovery with time**.** The number of myocytes in the LV of control and infarcted hearts was obtained by employing a methodology well-established in our laboratory [15-16]. The quotient between the number of myocytes present in the infarcted LVs and the number of LV myocytes in sham-operated animals gives the percentage of myocytes lost after infarction. The percentage of myocytes lost provides a quantitative measurement of infarct size, while the percentage of myocytes left correlates with ventricular function. The newly-formed myocardium in treated hearts was not included in this analysis, in order to evaluate the consequences of coronary ligature on infarct size independently of tissue reconstitution.

*Collagen accumulation in the spared LV.* Five-micrometer thick sections from the equatorial slice of the LV (see above) were stained with Masson’s trichrome and analyzed by optical microscopy (magnification 250X) in order to evaluate in the remote spared myocardium: (i) the volume fraction of myocytes, (ii) the volume fraction of fibrosis, and (iii) the numerical density and average cross-sectional area of fibrotic foci. Quantification of myocardial scarring resulting from focal myocyte cell loss was included in the analysis since foci may represent an important ongoing post-infarction tissue injury. According to a procedure previously described [17], for each section a quantitative evaluation of the volume fraction of myocytes and fibrotic tissue was performed in 60 adjacent fields from sub-endocardium, mid-myocardium and sub-epicardium. The measurements were obtained with the aid of a grid defining a tissue area of 0.16 mm^2^ and containing 42 sampling points each covering an area of 0.0038 mm^2^.

*Myocyte Cell Size.* In sections of 7 SO+V, 10 MI+V and 12 MI+GF hearts obtained from as many randomly selected TE rats, the cross-sectional area of myocytes was morphometrically determined by measuring the cell diameter, at the nucleus level, in transversally oriented myocytes**.** For each heart, 120 to 250 cellular measurements were performed.

**Immunohistochemical analysis**

Five-micrometer thick sections (see above), obtained from *****MI+GF and MI+V rat hearts***** treated with BrdC and exhibiting comparable infarct size, were analyzed to determine: (i) the expression and spatial distribution of connexin43 (Cx43) and N-cadherin, (ii) the incidence of c-kit+ CPCs, and (iii) the fraction of nuclei labeled by BrdU, in the infarcted, peri-infarcted and remote LV myocardium. The quantification of BrdU labeling in arterioles was not included in the study because the procedure does not allow us to assess the fraction of newly formed structures, which are constituted by a multiple layer of cells.

BrdU, c-kit, Cx43 and N-cadherin labeling was detected by immunofluorescence. LV sections were incubated with the primary antibody (monoclonal mouse anti-BrdU antibody, dilution 1:20, Dako, Glostrup, Denmark; rabbit polyclonal anti-c-kit antibody, dilution 1:20, Santacruz Biotecnology, Inc, Santa Cruz, CA, USA; mouse monoclonal anti-Cx43 antibody, dilution 1:250, Chemicon, Temecula, CA, USA; rabbit polyclonal anti-N-cadherin antibody, dilution 1:200, Calbiochem, San Diego, CA, USA)**.** Myocytes, smooth muscle cells and endothelial cells were identified by staining the same sections respectively with monoclonal mouse anti-α-sarcomeric actin antibody (anti-α-SARC; dilution 1:30, Dako), monoclonal mouse anti-α-smooth muscle actin antibody (anti-α-SMA; dilution 1:50, Dako), and polyclonal rabbit anti-vW factor (dilution 1:100, Dako) respectively. FITC-, TRITC- Cy5- conjugated anti-mouse or anti-rabbit secondary antibodies (Jackson Laboratory, Baltimore, PA, USA) were used to detect simultaneously the different epitopes. Nuclei were counterstained by DAPI (4',6-diamidino-2-phenylindole, Sigma Aldrich Italia, Milan, Italy).

The amount of newly formed myocardium was computed by calculating the number and size of small α-SARC+ myocytes according to a methodology previously employed [16]. The number of resistance arterioles was measured by counting vascular profiles labeled by α-SMA, within the infarcted myocardium*.*

**Electrophoretic and immunoblot analysis**

The infarcted and non-infarcted portions of the LV ventricles obtained from 5 MI+GF and 5 MI+V animals belonging to the MB group (Table S1) were weighed, frozen in liquid nitrogen and stored at -80 °C. For immunoblot assay of Cx43, the fresh ventricular tissue was lysed with 150-200 μL of lysis buffer containing the following (Sigma, St Louis, MO, USA): protease inhibitor dithiothreitol (0.5 mmol/L), DNase (50 μg/mL), RNase (50 μg/mL), HEPES (10 mmol/L), NaCl (400 mmol/L), EGTA (0.1 mmol/L), glycerol (5%), NP40 (3%). Equivalents of 100-125 μg of protein were separated by 10.5% SDS-PAGE, transferred on nitrocellulose filters and exposed to anti-Cx43 monoclonal mouse antibody (dilution 1:750, Chemicon). Binding sites were detected by peroxidase-conjugated specific anti-mouse IgG (dilution 1:2000, in ECL reagents; Amersham Biosciences**,** Piscataway, NJ, USA). The intensity of the bands representing the connexin protein was quantified using a densitometer (Molecular Dynamics, Sunnyvale, CA, USA) and the data were expressed as optical density units.

**Quantification of Kv (α) and accessory subunits by Real-Time RT-PCR**

*RNA isolation****.*** Immediately after death, the LV and RV were excised from the heart; each sample was frozen in liquid nitrogen, and stored at -80°C. Total RNA from each cardiac sample was isolated and DNase-treated with the RNeasy Fibrous Tissue Mini Kit (Qiagen, Milan, Italy) following the manufacturer's instructions. Isolated RNA was quantified (spectrophotometric absorbance at 260 nm) and purity was confirmed by the A260/A280 ratio. Integrity of total RNA was evaluated by ethidium bromide staining on denaturing agarose gels. RNA samples were stored at -80°C.

*Reverse transcription.* Single-stranded cDNA was performed by using High Capacity cDNA Reverse Transcription Kit (Applied Biosystems, Foster City, CA, USA) following the manufacturer's instructions. The reverse transcription reaction was performed by incubating a reaction mixture containing: 2 µg RNA, 10X RT-Buffer, 10X RT-random primers, 25X dNTP Mix (100 mmol/L), RNase inhibitor, and Multiscribe Reverse Transcriptase in a total of 20 µL 1X reaction buffer. Reverse transcription was performed at 25°C for 10 min, 37°C for 120 min and stopped by incubating at 85°C for 5 s.

*Real-Time RT-PCR*. Expression levels of Kv1.4, Kv4.2, Kv4.3 and KChiP2 genes were investigated using real-time quantitative RT-PCR and TaqMan® probe-based chemistry. When evaluated by the same method, the gene GAPDH showed stable expression in the LV and RV under all of the experimental conditions and was therefore used as endogenous control gene. Primers and probes were obtained from ABI (Applied Biosystems, Foster City, CA, USA) TaqMan Gene Expression Assay catalogue. These assays come in a 20X reaction mix, span an exon-exon junction, and are optimized to give approximately 100% efficiency. The real-time RT-PCR reactions were performed using TaqMan® Gene Expression Master Mix (Applied BioSystems) in a 20 µL reaction volume containing 50 ng of cDNA. All reactions were performed in triplicate and included a negative control. PCR reactions were carried out using an ABI Prism 7500 Sequence Detection System (Applied Biosystems). Cycling conditions were: 2 min at 50°C, 10 min at 95°C, and 40 cycles of 15 s at 95°C and 1 min at 60°C. Relative quantification of mRNA levels was obtained by the 7500 system software, which uses the comparative method (ΔCT).

**References**

1. Sgoifo A, Stilli D, Medici D, Gallo P, Aimi B, et al. (1996) Electrode positioning for reliable telemetry ECG recordings during social stress in unrestrained rats. Physiol Behav 60:1397-1401.
2. Martinez M, Calvo Torrent A, Pico Alfonso MA (1998) Social defeat and subordination as models of social stress in laboratory rodents: a review. Aggress Behav 24:241-256.
3. Walker MJA, Curtis MJ, Hearse DJ, Campbell RWF, Janse MJ, et al. (1988) The Lambeth Conventions: guidelines for the study of arrhythmias in ischaemia, infarction, and reperfusion. Cardiovasc Res 22:447-455.
4. Task Force of the European Society of Cardiology and the North American Society of Pacing and Electrophysiology (1996) Heart rate variability. Standards of measurement, physiological interpretation, and clinical use. Circulation 93:1043-1075.
5. Urbanek K, Rota M, Cascapera S, Bearzi C, Nascimbene A, et al. (2005) Cardiac stem cells possess growth factor-receptor systems that after activation regenerate the infarcted myocardium, improving ventricular function and long-term survival. Circ Res 97:663-673.
6. Macchi E, Cavalieri M, Stilli D, Musso E, Baruffi S, et al. (1998) High-density epicardial mapping during current injection and ventricular activation in rat hearts. Am J Physiol Heart Circ Physiol 275:H1886-H1897.
7. Taccardi B, Punske BB, Macchi E, MacLeod RS, Ershler PR (2008) Epicardial and intramural excitation during ventricular pacing: effect of myocardial structure. Am J Physiol Heart Circ Physiol 294:H1753-H1766.
8. Lapicque L (2007) Quantitative investigations of electrical nerve excitation treated as polarization. 1907. Biol Cybern 97:341-349.
9. Opthof T, Coronel R, Vermeulen JT, Verberne HJ, van Capelle FJ, et al. (1993) Dispersion of refractoriness in normal and ischaemic canine ventricle: effects of sympathetic stimulation. Cardiovasc Res 27:1954-1960.
10. Ogawa S, Furuno I, Satoh Y, Yoh S, Saeki K, et al. (1991) Quantitative indices of dispersion of refractoriness for identification of propensity to re-entrant ventricular tachycardia in a canine model of myocardial infarction. Cardiovasc Res 25:378-383.
11. Mitchell GF, Jeron A, Koren G (1998) Measurement of heart rate and Q-T interval in the conscious mouse. Am J Physiol Heart Circ Physiol 274:H747-H751.
12. Fuller MS, Sándor G, Punske B, Taccardi B, MacLeod RS, et al. (2000) Estimates of repolarization dispersion from electrocardiographic measurements. Circulation 102:685-691.
13. Lux RL, Gettes LS, Mason JW (2006) Understanding proarrhythmic potential in therapeutic drug development: alternate strategies for measuring and tracking repolarization. J Electrocardiol 39:S161-S164.
14. Dodge HT, Baxley WA (1969) Left ventricular volume and mass and their significance in heart disease. Am J Cardiol 23:528-537.
15. Olivetti G, Capasso JM, Meggs LG, Sonnenblick EH, Anversa P (1991) Cellular basis of chronic ventricular remodeling after myocardial infarction in rats. Circ Res 68:856-869.
16. Orlic D, Kajstura J, Chimenti S, Limana F, Jakoniuk I, et al. (2001) Mobilized bone marrow cells repair the infarcted heart, improving function and survival. Proc Natl Acad Sci U S A 98:10344-10349.
17. Stilli D, Lagrasta C, Berni R, Bocchi L, Savi M, et al. (2007) Preservation of ventricular performance at early stages of diabetic cardiomyopathy involves changes in myocyte size, number and intercellular coupling. Basic Res Cardiol 102:488-499.
