# Supplementary material for: Growth Factor-Induced Mobilization of Cardiac Progenitor Cells Reduces the Risk of Arrhythmias, in a Rat Model of Chronic Myocardial Infarction
Source: PLoS One. 2011 Mar 18;6(3):e17750. doi: 10.1371/journal.pone.0017750 (PMC3060871; doi:10.1371/journal.pone.0017750)
Supplement: Table S1 — Outline of the experimental protocols. (DOC) [file pone.0017750.s002.doc]

**Table S1. Outline** of the experimental protocols

| **In vivo studies** | **day** | **TE rats (n=60)** | **EM rats (n=43)** | **MB rats (n=30)** |
| --- | --- | --- | --- | --- |
|  |  |  |  |  |
| Chronic instrumentation for telemetry-ECG recording | 1 | + | - | - |
| **Telemetry-ECG recording in baseline conditions** | 7 | + | - | - |
| Myocardial infarction | 10 | + | + | + |
| Echocardiographic measurements | 32 | + (**selected subgroups)** | - | - |
| Telemetry-ECG recording during baseline and social stress conditions | 36 | + | - | - |
| Epicardial multiple-lead recording | 39 | - | + | - |
| Intramyocardial injection of HGF+IGF-1 or vehicle | 39 | + | + | + |
| Implantation of an osmotic pump for continuous (two weeks) BrdC delivery | 39 | + (**selected subgroups)** | - | - |
| Echocardiographic measurements | 52 | + (**selected subgroups)** | - | - |
| Telemetry-ECG recording during baseline and social stress conditions | 54 | + | - | - |
| Epicardial multiple-lead recording | 54 | - | + | - |
| Hemodynamic measurements |  | + (**selected subgroups)** | - | - |
| Sacrifice | 60 | + | + | + |
| **Post mortem studies** |  |  |  |  |
| Anatomical identification of myocardial infarction |  | + | + |  |
| Cardiac anatomy and infarct size |  | + | - | - |
| Morphometric analysis |  | + (**selected subgroups)** | - | - |
| Immunohistochemical analysis |  | + (**selected subgroups)** | - | - |
| Electrophoretic and immunoblot analysis |  | - | - | + (**selected subgroups)** |
| Quantitative RT-PCR measurements |  | - | - | + (**selected subgroups)** |
