# Supplementary material for: Growth Factor-Induced Mobilization of Cardiac Progenitor Cells Reduces the Risk of Arrhythmias, in a Rat Model of Chronic Myocardial Infarction
Source: PLoS One. 2011 Mar 18;6(3):e17750. doi: 10.1371/journal.pone.0017750 (PMC3060871; doi:10.1371/journal.pone.0017750)
Supplement: Table S2 — Echocardiographic data. (DOC) [file pone.0017750.s003.doc]

**Table S2. Echocardiographic measurements**

|  | **Pre-injection**  **SO (n=9)** | **MI (n=38)** |  | **Post-injection**  **SO+V (n=7)** | **MI+V (n=18)** | **MI+GF (n=19)** |
| --- | --- | --- | --- | --- | --- | --- |
| EF (%) | 89.5±0.9 | 82.4±1.2 * |  | 87.6±1.1 | 75.8±2.2 # | 76.5±2.3 # |
| FS (%) | 54.8±1.4 | 46.8±1.2 * |  | 52.2±1.5 | 40.5±2.0 # | 41.4±2.3 # |
| LVSD (mm) | 2.5±0.1 | 3.4±0.1 * |  | 2.7±0.1 | 3.8±0.2 # | 3.6±0.2 # |
| LVEDD (mm) | 5.5±0.2 | 6.3±0.2 * |  | 5.8±0.2 | 6.5±0.2 # | 6.1±0.2 |
| LVESV (µl) | 42±8 | 121±5 * |  | 44±5 | 145±19 # | 142±23 # |
| LVEDV (µl) | 397±43 | 625±44 * |  | 397±42 | 667±66 # | 547±51 |

Mean values±SE of echocardiographic parameters measured before GF/V injection (SO and MI groups) and 15 days after treatment (SO+V, MI+V and MI+GF groups). EF: ejection fraction; FS: fractional shortening; LVSD: left ventricular systolic diameter; LVEDD: left ventricular end-diastolic diameter; LVESV: left ventricular end-systolic volume; LVEDV: left ventricular end-diastolic volume. *p<0.01 significant differences between SO and MI; # p< 0.01 significant differences vs. SO+V.
