# Supplementary figures and images for: Growth Factor-Induced Mobilization of Cardiac Progenitor Cells Reduces the Risk of Arrhythmias, in a Rat Model of Chronic Myocardial Infarction
Source: PLoS One. 2011 Mar 18;6(3):e17750. doi: 10.1371/journal.pone.0017750 (PMC3060871; doi:10.1371/journal.pone.0017750)

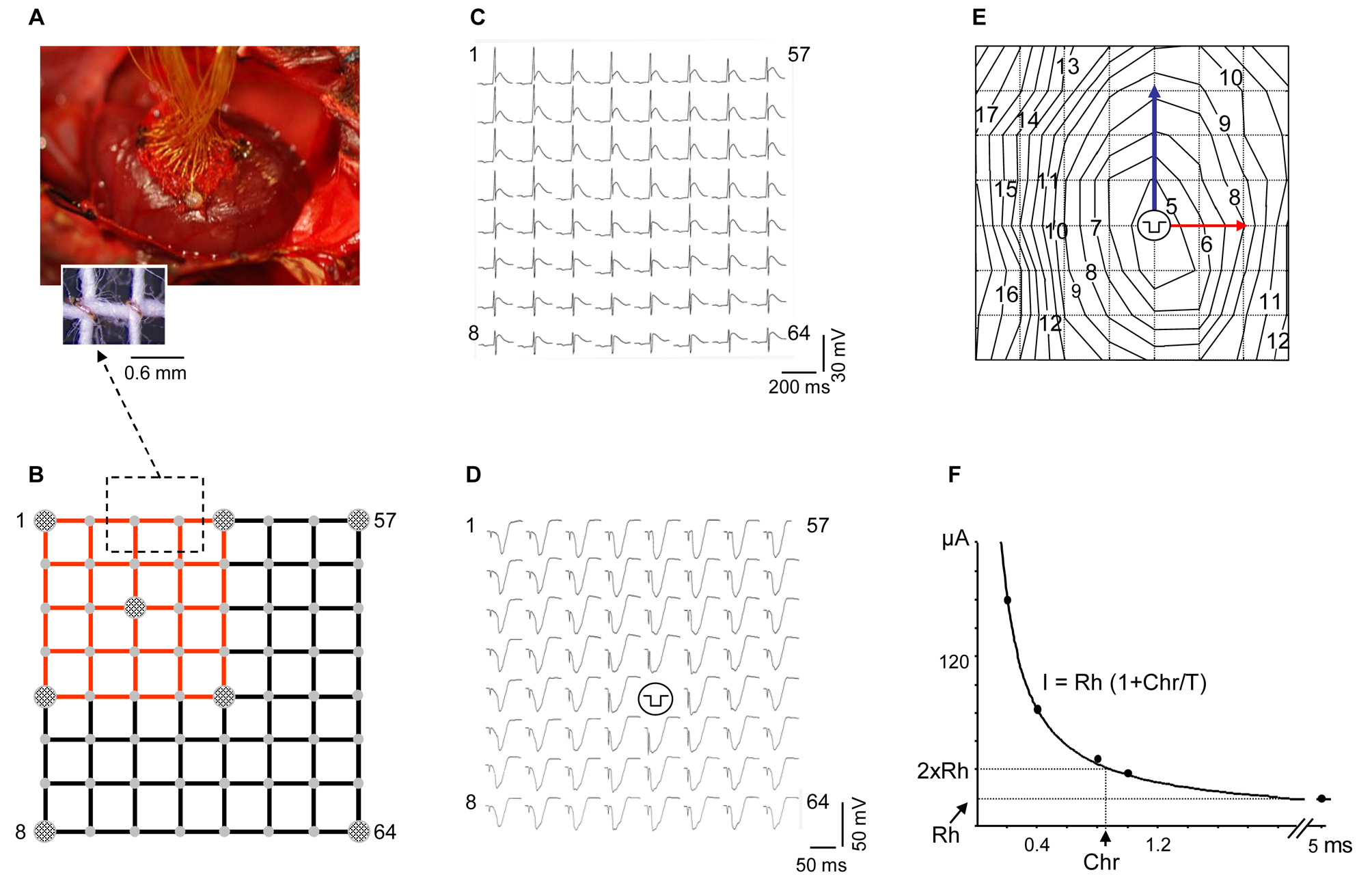

Supplement: Figure S1 — Epicardial multiple-lead recording: electrode arrays and procedures. (A) Electrode array positioned on the anterior aspect of the ventricular surface. (B) Schematic representation of the 5×5 (in red) and 8×8 electrode arrays showing the positions (larger circles) of the 5 and 8 selected electrodes used for specific pacing protocols. (C–D): Unipolar electrograms collected by means of the 8×8 electrode array during normal sinus rhythm (C) and ventricular pacing at the electrode indicated by the pulse symbol (D). In (E), example of paced activation isochrone map used for computing conduction velocity longitudinally (blue-arrow) and transversally (red-arrow) to fiber orientation; numbers on each isochrone line indicate the activation time in ms. In (F), strength-duration curve obtained in a control rat by plotting pulse threshold current I as a function of pulse duration T (Rh: Rheobase, Chr: Chronaxie). (TIF) [file pone.0017750.s004.tif]
